# Comparative Analysis of White and African American Groups Reveals Unique Lipid and Inflammatory Features of Diabetes

**DOI:** 10.1007/s40615-025-02642-z

**Published:** 2025-09-26

**Authors:** Gabriela Pacheco Sanchez, Miranda Lopez, Leandro M. Velez, Ian Tamburini, Naveena Ujagar, Julio Ayala Angulo, Gabriela De Robles, Hannah Choi, John Arriola, Rubina Kapadia, Alan B. Zonderman, Michele K. Evans, Cholsoon Jang, Marcus M. Seldin, Dequina A. Nicholas

**Affiliations:** 1 Department of Molecular Biology and Biochemistry, Charlie Dunlop School of Biological Sciences, University of California, Irvine, CA, USA; 2 Department of Biological Chemistry, and Center for Epigenetics and Metabolism, School of Medicine, University of California, Irvine, CA, USA; 3 The Laboratory of Epidemiology and Population Sciences, National Institute On Aging, National Institutes of Health, Maryland, USA

**Keywords:** Lipidomics, Inflammation, Diabetes, Health disparities

## Abstract

Diabetes is a metabolic and inflammatory disease that disproportionately affects African American populations, yet clinical diagnostics often rely on biomarkers discovered and validated predominantly in White cohorts. This study investigates race-specific lipid and inflammatory features of diabetes to uncover biologically distinct disease signatures that may contribute to disparities in diagnosis and management. We analyzed clinical parameters from a well-matched subset of the HANDLS cohort (*N* = 40) and conducted targeted plasma lipidomics and multiplex cytokine profiling across African American and White individuals from the HANDLS cohort with and without diabetes. Then we validated key findings using a large and diverse cohort of African American and White individuals with type 2 diabetes from the NIH AllofUs program (*N* = 17,339). Our results reveal racially divergent signatures of diabetes. White individuals with diabetes exhibited elevated Cholesterol:HDL ratios, triglycerides, and classical inflammatory markers such as hs-CRP. In contrast, African American individuals with diabetes displayed minimal lipid elevations but showed increased Th17-related cytokines1. These differences were independent of statin use, age, and body mass index. Additionally, correlations between lipid to cytokine ratios and the glycemic marker hemoglobin A1C differed sharply by race, suggesting that the pathophysiology of diabetes is not uniform across populations. Our findings challenge standard diabetes biomarkers and emphasize the need for more inclusive diagnostic frameworks. By identifying population-specific biological patterns of diabetes, this study provides important insight into the roots of persistent health disparities and underscores the value of precision approaches to equitable diabetes care.

## Introduction

Diabetes affects an estimated 38.4 million individuals in the USA—approximately 11.6% of the population—with disproportionately high prevalence among individuals from historically marginalized racial and ethnic groups, including African Americans [1–3]. While race is a social construct and not a biological determinant, it reflects lived experiences, including exposure to structural racism, chronic psychosocial stress, disparities in healthcare access, and environmental influences—all of which can shape biological outcomes [4–6]. These race-associated factors may contribute to variation in immune and metabolic pathways, influencing how diabetes manifests and progresses across populations. The pathophysiology of diabetes is characterized by substantial heterogeneity, with genetic variation contributing to diverse disease mechanisms and clinical presentations across populations [7]. Polygenic risk scores have revealed population-specific genetic architectures that influence disease susceptibility, progression, and treatment response [8, 9]. The impact of genetic variation is likely a large contributor to differences in the presentation of type 2 diabetes across racial and ethnic populations.

Clinical measurements of glucose management like hemoglobin A1C (HbA1C) and measurements of insulin resistance, the homeostatic model assessment of insulin resistance (HOMA-IR) are preferably used in the current clinical assessment of diabetes [10–12]. HbA1C, reflecting average glycemic control over the preceding 2–3 months through non-enzymatic glycation of hemoglobin, serves as both a diagnostic criterion and a predictor of diabetic complications [13, 14]. HOMA-IR, calculated from fasting glucose and insulin levels, provides a validated surrogate measure of insulin sensitivity and pancreatic β-cell function, capturing the dynamic interplay between insulin secretion and peripheral insulin action [15]. In addition to disrupted glucose homeostasis which is routinely assessed, presentation of diabetes is also distinctively characterized by dysregulation of lipid metabolism (dyslipidemia) and chronic inflammation [16–18]. Efforts to study diabetes-related metabolic phenotypes in diverse populations have provided insights into the characterization of lipid profiles and inflammatory markers in populations with diabetes [19]. Further, reports have historically found an increase in systemic markers such as C-reactive protein (CRP), and the pro-inflammatory cytokines interleukin 6 (IL-6) and tumor necrosis factor alpha (TNF-α) as features of diabetes [20–22]. More recent literature in type 2 diabetes (T2D) research has discovered an elevation of Th17 cytokines (IL-17A, IL-17E, IL-17F, IL-21, and IL-22) in people with T2D. Particularly, IL-17A has been reported to be elevated in patients with T2D compared to patients without T2D [23–25]. Despite these findings, most biomarker discovery studies have not been designed to assess how these immunometabolic features vary across racial or ethnic groups.

Studies in the field of cardiometabolic disease provide evidence to support that disparities persist regarding clinical features of diabetes in diverse populations. For example, disparate levels of lipids (cholesterol, high-density lipoprotein (HDL), low-density lipoprotein (LDL), and triglycerides) have been reported in African Americans compared to White individuals in the context of cardiometabolic disease [26, 27]. Yet there is a dearth of studies that have systematically explored how these differences intersect with immune activation in diabetes.

Our study fills this gap by characterizing race-specific lipid and inflammatory phenotypes in well-matched groups of African American and White adults with and without diabetes. Given the complex relationships between inflammation and lipid metabolism in diabetes, we employed a novel exploratory approach examining cytokine-to-lipid ratios to capture potential immune regulation of systemic lipid metabolism and its association with glycemic control. By leveraging detailed clinical, lipidomic, and cytokine profiling data in a diverse HANDLS cohort and validating the main findings in a large AllOfUs cohort, we aim to uncover population-specific disease signatures that may inform more equitable diagnostic and therapeutic strategies.

## Research Design and Methods

### HANDLS Population

For the in-depth independent and integrated biological analysis of clinical parameters, lipidomics, cytokine profiling, and immunological phenotyping, we used a subcohort from the Healthy Aging in Neighborhoods of Diversity across the Life Span (HANDLS) study (Suppl. Fig S1) [28]. Study participants were sampled from Wave 1 of this study. Based on published studies identifying *N* = 10 as sufficiently powered to detect cytokine differences in humans with type 2 diabetes [23–25], *N* = 40 participants were randomly selected and divided into four matched comparison groups (*N* = 10 per group): White individuals without diabetes (NoDx-White), White individuals with diabetes (Dx-White), African American individuals without diabetes (NoDx-AA), and African American individuals with diabetes (Dx-AA). These groups were equally distributed by race, diabetes status, and sex, with each group matched by age, body mass index (BMI), and poverty status (Table 1). To minimize the impact of comorbidities, the exclusion criteria for the HANDLS subcohort included patients ever diagnosed with Alzheimer’s disease, rheumatoid arthritis, ankylosing spondylitis, cancer, asthma, or psoriasis.

Based on the absence of insulin use (36/40), 90% of the cohort had confirmed T2D. Dietary intake of lipids was determined using the USDA Automated Multiple Pass Method of dietary recall, which is an interviewer-administered computerized method for collecting 24-h dietary recalls [29].

### Clinical Parameters Evaluated in HANDLS Subcohort

To define the clinical features characteristic of diabetes in White and African American participants, we performed a univariate comparison of clinical parameters. We evaluated waist hip ratio (WHR), cholesterol (Chol) levels, high-density lipoprotein (HDL), cholesterol to HDL ratio (CholHDLRat), low-density lipoprotein (LDL), very low-density lipoprotein (VLDL), triglycerides, HbA1C, insulin, fasting glucose, and high-sensitivity C-reactive protein (hs-CRP).

### Targeted Lipidomics Using Liquid Chromatography Mass Spectrometry (LC-MS) in HANDLS Subcohort

#### Metabolite Extraction

To extract metabolites from plasma samples, 300μL −20 °C 1000:1 isopropanol:lipidomics standard (extraction solvent) was added to 10μL of aliquoted plasma sample and incubated on ice for 10 min, followed by vortexing and centrifugation at 15,800 × g for 15 min at 4 °C. 100 μL of the clear supernatant (extract) was transferred to a glass mass spectrometry vial.

#### LC-MS

Plasma extracts were analyzed by LC-MS. Metabolites were analyzed using a quadruple-orbitrap mass spectrometer (Q-Exactive Plus Quadrupole-Orbitrap, Thermo Fisher) coupled to reverse-phase ion-pairing chromatography. The mass spectrometer was operated in positive ion mode with resolving power of 140,000 at m/z 200 and scan range of m/z 290–1200. The LC method utilized an Atlantis T3 column (150 mm × 2.1 mm, 3 μm particle size, 100 Å pore size, Waters) with a gradient of solvent A (90:10 water: methanol with 1 mM ammonium acetate and 35 mM acetic acid) and solvent B (98:2 isopropanol: methanol with 1 mM ammonium acetate and 35 mM acetic acid). The LC gradient was 0 min, 25% B, 0.150 mL/min; 2 min, 25% B, 0.15 mL/min; 5.5 min, 65% B, 0.150 mL/min; 12.5 min, 100% B, 0.150 mL/min; 16.5 min, 100% B, 0.150 mL/min; 17 min, 25% B, 0.150 mL/min; and 30 min, 25% B, 0.150 mL/min. Other LC parameters were column temperature 45 °C, autosampler temperature was set to 4 °C, and the injection volume of the sample was 3 μL. Lipidomics data analysis was performed with Compound Discover and MAVEN software.

### Cytokine Profiling Using Luminex Platform in HANDLS Subcohort

#### Plasma Samples

Ten microliters of plasma (undiluted) was evaluated in a 384-well plate for cytokine profiling. We used the MILLIPLEX^®^ MAP human kits Cytokine/Chemokine/Growth Factor Panel (Millipore Cat# HCYTA-60 K-PXBK48) and the Th17 5-plex (IL21, IL23, IL31, IL33, and MIP-3a) (Millipore Cat# HTH17MAG-14 K) to assay 53 cytokines per sample. All reagents were used at 10 <L to adjust to 384 well format. Samples were read using a xMAP INTELLIFLEX^®^ System (Luminex). Belysa^®^ Immunoassay Curve Fitting Software (Millipore) was used for curve fitting.

### Immune Phenotyping of Cellular Populations Using Flow Cytometry in HANDLS Subcohort

#### Flow Cytometry

Leukocyte populations were phenotyped in PBMCs with 23 markers (Suppl. Table 11). All staining steps were performed at 4 °C protected from light. PBMCs were stained with live/dead stain Zombie NIR for 20 min. PBMCs were washed with FACS buffer (PBS + 0.1% BSA + 2 μM EDTA) and centrifuged at 500 g for 5 min. Supernatant was removed, and 25 μL of Human TruStain FcX was added for 10 min. Next, 25 μL of surface antibody master mix diluted in BD Biosciences Brilliant Stain Buffer (CAT# 566,349) was added to PBMCs for 20 min. PBMCs were then washed with FACS buffer, centrifuged, and supernatant removed. PBMCs were fixed with Biolegend’s Fixation Buffer (CAT# 420,801) for 20 min. PBMCs were washed with Biolegend’s Intracellular Staining Permeabilization Wash Buffer (CAT# 421,002) twice followed by addition of 25 μL of antibody master mix diluted in Biolegend’s Intracellular Staining Permeabilization Wash Buffer for 20 min. PBMCs were washed with Biolegend’s Intracellular Staining Permeabilization Wash Buffer and resuspended in 200 μL of 1% paraformaldehyde diluted in PBS pH 7.4 for acquisition using the spectral flow cytometer Cytek’s 3-laser Northern Lights. Data was analyzed with FlowJo v.10.

### Statistical Analysis Using the ANOVA Model in HANDLS Subcohort

Univariate analysis was performed using two-way ANOVA and post-ANOVA comparisons using Fisher’s least significant differences (LSD) test. Two-way ANOVA and post-ANOVA multiple comparisons were performed using R Studio and GraphPad Prism. To control for multiple testing, all *p*-values were adjusted using the False Discovery Rate (FDR) correction method, with statistical significance defined as FDR-adjusted *p* < 0.05.

Briefly, all datasets (clinical parameters, dietary intake data, targeted lipidomics, plasma cytokines, and immune phenotyping) were assessed for normal distribution. Then, for targeted lipidomics and plasma cytokines, we performed a box-cox transformation on the datasets as needed to correct for heteroscedasticity and to better satisfy the normality assumption of the two-way ANOVA model. The two-way ANOVA is a statistical procedure to estimate differences in the means of a dataset by two variables. In this work, we used two-way ANOVA to fit a model considering race (White/African American), disease status (diabetes or no diabetes), and the interaction between the two as our factors. The interaction term in the ANOVA model investigates whether the effects of disease on the means evaluated (clinical parameters, lipids, and inflammatory markers) differ by race. These hypotheses are tested by the protected LSD procedure where we first look at the significance of the overall F-test, whether there is a difference in mean across any of the four groups of participants (divided in 4 by disease and race). We then look at the four pre-specified group pairwise differences in the mean clinical parameters of interest and judge their significance by a t-test with a pooled standard error. The four pre-specified group pairwise differences we evaluated were: NoDx-White vs Dx-White, NoDx-AA vs Dx-AA, NoDx-White vs NoDx-AA, and Dx-White vs Dx-AA. All graphics were generated using GraphPad Prism v.10.

### Variability and Clustering Analysis Using Principal Component Analysis (PCA), K-Means, and Gap Statistics in the HANDLS Subcohort

Principal Component Analysis (PCA) is a bioinformatics and statistical tool used to reduce data dimensionality into principal components (PC1, PC2, etc.) while maximizing the variance captured in the first components. PCA identifies which factors correlate with each other and determines their relative contributions to dataset variability [30]. In this study, PCA was applied to identify the main variables responsible for variability in the clinical parameters of the HANDLS subcohort [30].

K-means is an unsupervised machine learning algorithm that partitions datasets into a specified number (K) of clusters, while gap statistics is a complementary tool used to determine the optimal number of clusters for a given dataset [31]. In this work, *K*-means clustering combined with gap statistics was used to partition the lipid and inflammatory biomarker datasets into meaningful clusters, thereby reducing the number of analytes into manageable groups for analysis.

### Feature Selection Analysis Using Orthogonalized Partial Least Squares Discriminant Analysis (OPLS-DA) in HANDLS Subcohort

OPLS-DA is an iteration of the supervised clustering approach partial least squares discriminant analysis (PLS-DA) [32–34]. OPLS-DA generates latent variables (LVs) that are analogous to the principal components obtained by PCA but constrained by categorical information. OPLS-DA applies orthogonal rotations to the analysis to obtain maximum separation of classes along the LV1 axis; hence, a single LV serves as a predictor for the class, while other components describe the variation orthogonal to the first predictive component (LV1). OPLS-DA was performed using the Solo eigenvector research software. We limited the application of this tool to build a feature selection model using our dataset as a calibration set only, an approach conducted in previously published work [35, 36]. Each dataset was *z*-scored before upload to the Solo software. Cross-validation was performed using the leave-one-out strategy. Performance of the feature selection model generated was evaluated by statistics *R*^2^ Cal or *R*^2^ calibration. A higher *R*^2^ Cal value indicates a better fit of the model.

### Correlative Analysis of Lipid to Cytokine Ratios to Clinical Markers of Diabetes in HANDLS Subcohort

The concept of using ratios of circulating biomarkers has been established as an emerging approach in biomarker discovery, particularly in chronic diseases such as Alzheimer’s disease and in genomics applications [37, 38]. However, in the field of metabolic diseases such as diabetes, cytokine-to-lipid ratios remain exploratory. For this study, we employed this novel approach to test lipid to cytokine ratios as an alternative method for studying the intersection of immunological (cytokines) and metabolic (lipids) pathways in diabetes, hypothesizing that these ratios might provide more reproducible biomarkers than individual measurements alone.

The ratio of every possible lipid to cytokine combination was computed for all subjects of the HANDLS subcohort (*N* = 40). The ratios and their significance were generated using the corAndPvalue() function from the WGCNA package (version 1.72-5) in R Studio (version 4.2.1). Further, we evaluated which lipid to cytokine ratios uniquely correlated to HbA1c and HOMA-IR in each group. We filtered for lipid to cytokine ratios that correlated significantly (*p*-value < 0.05) with HbA1C and HOMA-IR in at least one racial group. We plotted correlation statistics for these ratios for White and African American individuals using ggplot2 (version 3.5.1).

### AllofUs Population Study and Experimental Design

For validation of the main clinical parameters findings in the HANDLS subcohort, we used the large, diverse, and multi-site AllofUs study (Suppl. Fig S1) [39]. We accessed the AllofUs researcher workbench and generated a dataset of 17,339 participants, African American and White individuals with T2D without complications (Registered Tier Data v.7). We selected the cohort based on disease status (T2D) and self-reported race. All participants that were diagnosed with T2D within the AllofUs study were selected if they fell within an age range of 30 to 65 years old and BMI range of 20 to 42, given these are the same ranges for the HANDLS cohort. Custom SQL queries were used to extract and export data to Rstudio. We computed descriptive statistics for each clinical biomarker evaluated (cholesterol, cholesterol/HDL ratio, triglycerides, insulin, glucose, and CRP) and presented them using the kable package. We adjusted for BMI and age while accounting for race by fitting linear models to each biomarker. Results were visualized using ggplot2 and ggpubr. Data was not adjusted by sex or poverty status.

The institutional review board of the National Institute of Environmental Health Sciences and the National Institutes of Health approved these protocols. The University of California Irvine Institutional Review Board exempted this study from review.

## Results

### Clinical Lipids Are Major Drivers of Variability in the HANDLS Subcohort

**Fig. 1:**
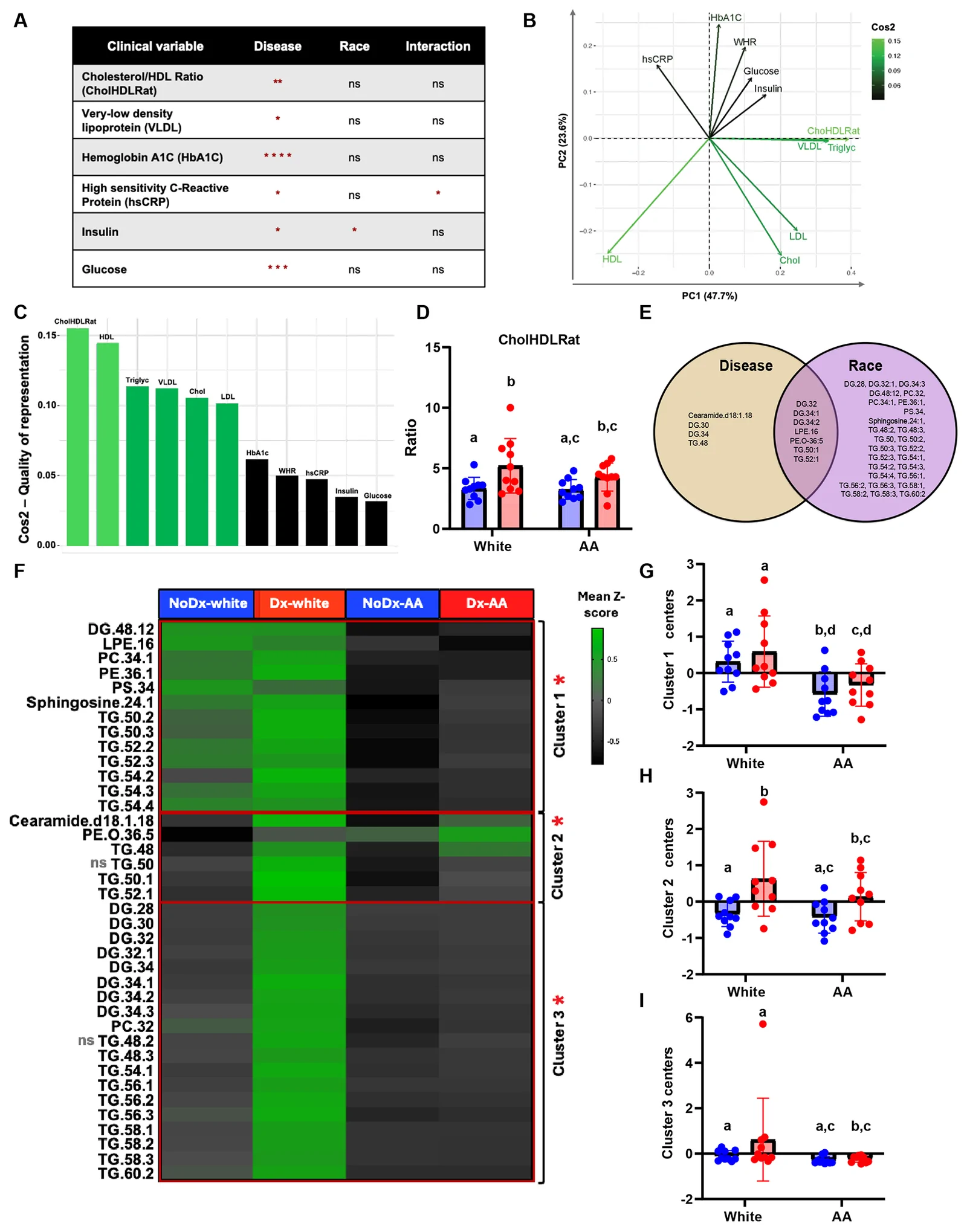
Lipids characterize diabetes in White groups but not in AA groups in a diverse HANDLS subcohort. **A** Table showing clinical parameters that differed statistically based on disease (no diabetes vs diabetes), race (White vs AA), and on the interaction of both variables (ns = not significant, * = *p*-value < 0.05, ** = *p*-value < 0.01, *** = *p*-value < 0.001, and **** = *p*-value < 0.0001). **B** Principal Component Analysis (PCA) showing correlations among clinical parameters evaluated and Cos2 color gradient indicating the quality of representation of clinical parameters of PCA from lowest to highest (black to lightest green). **C** Contribution bar chart displaying the order of parameters contributing to variability from highest to lowest (highest light green bar to lowest black bar) based on Cos2. **D** Bar/dot graph showing results from multiple statistical comparisons of Cholesterol/HDL ratio (CholHDLRat) among White and AA with and without diabetes. **E** Venn diagram showing 38 lipids that were significantly modulated based on disease (no diabetes vs diabetes), race (White vs AA), and on both disease and race. **F** Heatmap showing mean z-score value per comparison group from lowest (dark grey) to highest (light green) lipid species evaluated univariately and through cluster analysis (clusters 1, 2, and 3) generated using *K*-means and gap statistics. “ns” next to TG.50 and TG.48.2 represent non-significance in post-anova comparisons and red asterisks next to clusters represent statistically significant clusters. **G** Bar/dot graph showing results from post-anova multiple comparisons of lipid cluster 1 among White and AA with and without diabetes. **H** Bar/dot graph showing results from post-anova multiple comparisons of lipid cluster 2 among White and AA with and without diabetes. **I** Bar/dot graph showing results from post-hoc multiple comparisons of lipid cluster 3 among White and AA groups with and without diabetes. X axis represents cluster center measurements. Blue = People without diabetes. Red = people with diabetes. Statistical analysis performed using Two-way ANOVA with Box Cox transformed values followed by Fisher’s LSD post-comparison test (unadjusted p-values). Statistical post-hoc comparisons were performed only between matched groups based on diabetes status and race, and comparisons between persons with and without diabetes were not included in the analysis. *p*-values obtained from multiple post-hoc comparison analysis are represented using a statistical letter system, where significantly different *p*-values are represented by different letters and non-significant *p*-values are represented by the same letters

To define the clinical features characteristic of diabetes in White and African American participants, we performed a univariate comparison of the clinical parameters related to diabetes available in the HANDLS subcohort. First, we performed a two-way ANOVA to determine whether diabetes status, race, and/or the interaction of both variables can modify the clinical parameters in our cohort (Fig. 1A). We found that CholHDLRat, VLDL, HbA1C, hs-CRP, insulin, and glucose levels were significantly modulated by diabetes status. Insulin was the only parameter that was significantly modulated by race in our statistical model (Fig. 1A). Though insulin was not significantly different in any individual comparisons (Suppl. Fig. S2A), insulin levels were significantly different between NoDx-White and Dx-White when adjusted for insulin use (Suppl. Fig. S2B). hs-CRP was the only parameter that was significantly modulated by the interaction of disease and race. Confirming our statistical model, HbA1C and fasting glucose were significantly different between individuals with and without diabetes in each racial group (Suppl. Fig. S3).

To determine the sources of variation in the clinical parameters evaluated in the HANDLS subcohort, we performed PCA (Fig. 1B, C). By projecting the variables on principal component 1 (PC1), we determined that clinical lipid measurements like CholHDLRat, HDL, and triglycerides were the top contributors to variability (Fig. 1C). HbA1C, fasting glucose, and insulin contributed the least to the variability evaluated in our data. Alternatively, CholHDLRat, the main driver of variability, was only significantly different when compared between individuals with and without diabetes in the White group, even after adjusting for statin use (Fig. 1D and Suppl. Fig. S4A). From these data, we conclude that variability in diabetes-associated clinical parameters appears primarily driven by select clinical lipid measurements, particularly cholesterol-to-HDL ratio (CholHDLRat), HDL cholesterol, and triglycerides. Additionally, the significant CholHDLRat differences observed exclusively in the White group suggest that this variability is influenced by both disease status and racial background.

### Plasma Lipidomes Characterize Diabetes in White but Not in African American Groups in the HANDLS Subcohort

Given that lipids are main drivers of variability in the dataset, we first evaluated the dietary intake of lipids. Only some differences in short-chain fatty acids between NoDx-White and NoDx-AA were observed when comparing lipid intake (Suppl. Table S2). Next, we performed targeted plasma lipidomics to identify specific endogenous lipids that could be differentially abundant across our comparison groups. We performed two-way ANOVA to assess whether disease, race, and/or both variables significantly modulated the differences in lipid abundance seen in our HANDLS subcohort. We found that 38 lipids were significantly modulated in a model where disease, race, or both variables were evaluated (Suppl. Table S3 and Fig. 1E). The majority of these lipids were triglycerides (Fig. 1F). We next performed multivariate analysis using *K*-means and gap statistics on the significantly modulated lipids. By comparing cluster centers, a measurement that represents the average expression of all correlated lipids in one cluster, we found that all 3 clusters generated were significantly different in at least one comparison performed (Fig. 1F). Cluster 1, comprising mainly polyunsaturated long-chain triglycerides (TG) phosphatidylcholine (PC), phosphatidylethanolamine (PE), and sphingosine, was increased in White individuals over the African American group regardless of diabetes status (Fig. 1G). Cluster 2 was increased in Dx-AA and Dx-White compared to NoDx-AA and NoDx-White, respectively (Fig. 1H). Cluster 3, composed mainly of long-chain diacylglycerides (DG) and very long-chain TG, was increased in Dx-White compared to Dx-AA (Fig. 1I). We conclude that long and very long-chain DGs and TGs are most abundant in and most impacted by diabetes status in the White participants.

### Classical Measures of Inflammation Characterize Diabetes in White but Not in African American Groups in HANDLS Subcohort

**Fig. 2:**
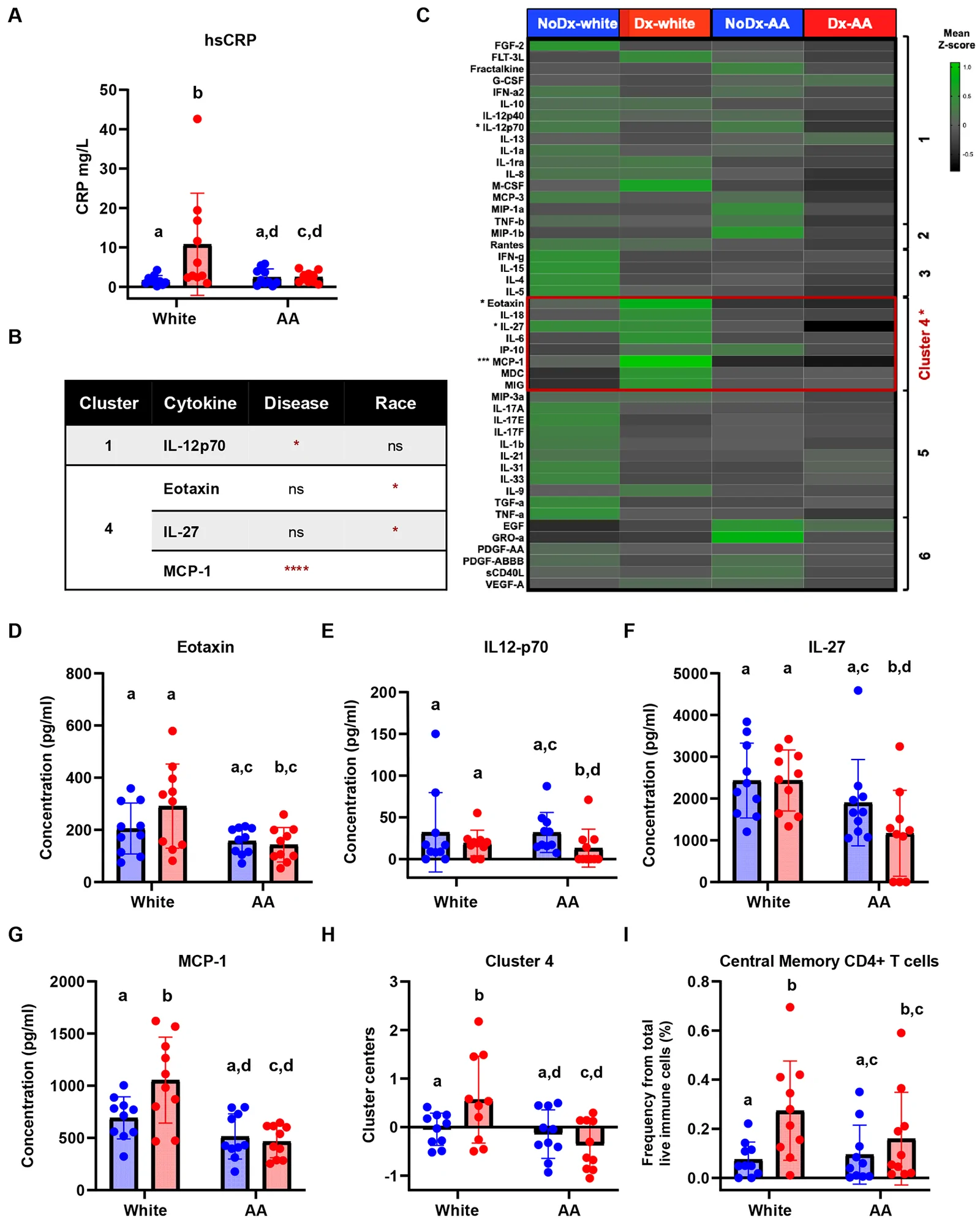
Plasma inflammatory profiles characterize diabetes in White but not in AA groups in a diverse HANDLS subcohort. **A** Bar/dot graph showing results from multiple statistical comparisons of high sensitivity C-reactive protein (hsCRP) among White and AA with and without diabetes. **B** Table showing cytokines and their respective clusters that differed statistically based on disease (no diabetes vs diabetes), race (White vs AA) or interaction of the variables (ns = not significant, * = p-value < 0.05, and **** = *p*-value < 0.0001). **C** Heatmap displaying mean z-score value per comparison group of plasma cytokines evaluated and per cytokine cluster (generated by K-means and Gap statistics analysis). Asterisks next to cytokines and clusters of cytokines represent significantly modulated clusters. **D-G** Bar/dot graph showing cytokines **D** eotaxin, **E** IL-27, **F** IL12p-70, and **G** MCP-1 which were statistically different among comparison groups. **H** Bar/dot graph showing results from statistical comparison of cytokine cluster 4 among White and AA with and without diabetes. **I** Bar/dot graph showing results from statistical comparison of cellular population central memory CD4 + T cells among White and AA with and without diabetes. The X axis represents cluster center measurements. Blue = people without diabetes, Red = people with diabetes. Statistical analysis performed using two-way ANOVA with Box-Cox transformed values followed by Fisher’s LSD post-comparison test (unadjusted p-values). Statistical post-ANOVA comparisons were performed only between matched groups based on diabetes status and race. Comparisons between persons with and without diabetes were not included in the analysis. *p*-values obtained from multiple post-ANOVA comparison analyses were represented using a statistical letter system, where significantly different *p*-values are represented by different letters and non-significant *p*-values are represented by the same letters

hs-CRP, the most common clinical inflammatory marker [40–42], was not a significant contributor to variability in our dataset (Fig. 1B and 1, C). Further, despite hs-CRP being significantly modulated in a model in which diabetes status and race were interactive variables (Fig. 1A), multiple comparison analysis showed that hs-CRP was only elevated in diabetes in the White group, even after adjusting for statin use (Fig. 2A, Supp. Fig. S4B). Therefore, we measured other systemic inflammatory biomarkers that could better characterize inflammation in this African American cohort.

We performed multiplex cytokine and growth factor profiling using the Luminex platform. We probed for 53 analytes in the plasma. After quality control, we obtained concentration values for 47 molecules. Our initial statistical model assessment and univariate multiple comparisons among the 4 groups indicated that two cytokines were significantly modulated by disease status, IL-12p70 and MCP-1, and two other cytokines by race, eotaxin and IL-27 (Fig. 2B, C). Notably, all 4 cytokines (eotaxin, IL-27, IL12-p70, and MCP-1) were increased in Dx-White when compared to Dx-AA (Fig. 2D-–G). Additionally, IL-27 and IL-12p70 were decreased in Dx-AA when compared to NoDx-AA and MCP-1 was increased in Dx-White compared to NoDx-White (Fig. 2E-–G). Because cytokine production has a high probability of covariance, we performed K-means and gap statistics that generated 6 clusters. By comparing cluster centers, we determined that cluster 4, which included eotaxin, IL-27, and MCP-1, was significantly increased in Dx-White compared to all other groups (Fig. 2C, H). We conclude that systemic levels of specific cytokines eotaxin, IL-27, and MCP-1 could account for differences in diabetes inflammatory status among diverse populations.

Due to the lack of a classical diabetes-associated inflammatory profile (i.e. IL-6, TNF-α, IL-1b, and hs-CRP) in the plasma of the African American cohort (Fig. 2A and Suppl. Fig. S5), we analyzed immune cell populations to determine if specific immune cell types could be contributing to this difference in cytokine profiles. Using flow cytometry, we found that central memory CD4^+^ T cells, a population of immune cells reported to play a modulatory role in diabetes [43, 44], were significantly increased only in Dx-White and not in Dx-AA (Fig. 2I, Supp. Fig. S6). No other cell populations in human blood were significantly different. We conclude that in our HANDLS subcohort, immune cell populations reported in the literature to have changes in frequencies in diabetes only characterize disease in White and not in the African American cohorts.

### Elevated Lipids and Classical Inflammatory Markers Are Features of Diabetes in the White Group While Th17 Inflammatory Features Characterize Diabetes in the African American Group in the HANDLS Subcohort

**Fig. 3:**
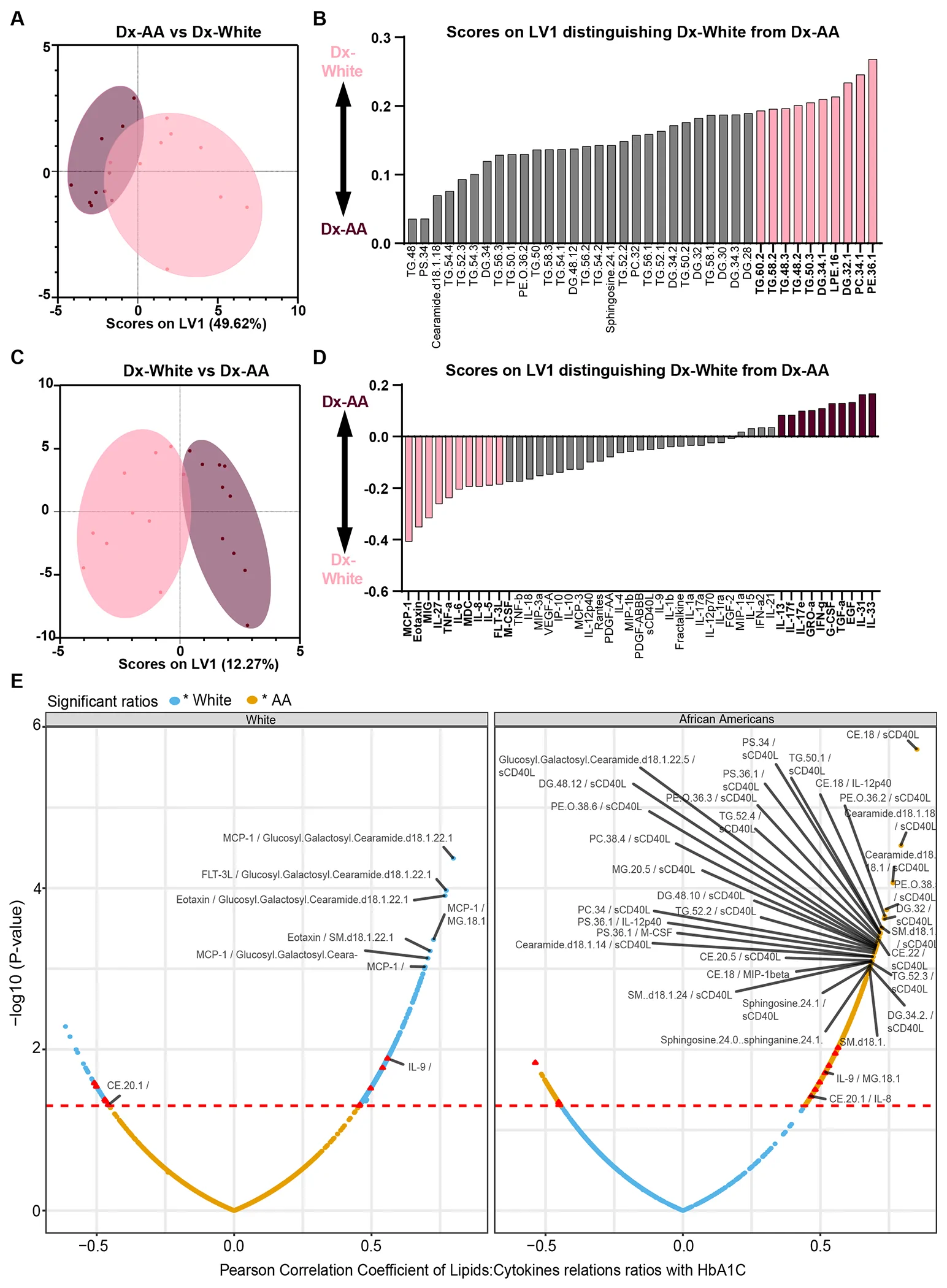
Lipid and inflammatory features characteristic of diabetes in a diverse HANDLS subcohort. **A** Orthogonal partial least squares discriminant analysis (OPLS-DA) plot of lipid features correlated with the presentation of diabetes in White (pink circle) and AA (burgundy circle) cohorts. **B** Bar graph plot displaying scores on LV1 showing lipids that distinguish diabetes in the White cohort. The top 10 correlated features to diabetes in the White cohort are highlighted in pink. **C** OPLS-DA plot of inflammatory features correlated with the presentation of diabetes in White (pink circle) and AA (burgundy circle) groups. **D** Bar graph plot displaying scores on LV1 which show inflammatory characteristic features of diabetes in White (pink bars) and AA (burgundy bars) groups. The top 10 correlated features to diabetes in White and AA groups are highlighted in pink and burgundy, respectively. *X*-axis represents scores on latent variable (LV) 1. *Y*-axis represents scores on LV2 not used for analysis. **E** Volcano plots showing all permuted lipid:cytokine correlations with HbA1C in White and AA. Briefly, all possible ratios were calculated for each lipid:cytokine, correlated to HbA1C, and subset for all correlations that were significant in each racial group only or in both. Correlations uniquely significant in White are colored in blue and correlations uniquely significant in AA are colored in yellow. Significant correlations in both groups are represented by red triangles. *X*-axis indicates the Pearson correlation coefficient. *Y*-axis indicates the −log10 of *p*-values for the lipid/cytokine ratio correlated to HbA1C. The dotted red line represents the threshold of significance values, above *p*-value < 0.05 and below *p*-value > 0.05

Unable to detect markers of inflammation specific to Dx-AA, we next used OPLS-DA to identify lipids and inflammatory features that characterize diabetes in both White and African American cohorts. By performing feature selection with OPLS-DA using the set of 38 significantly modulated lipids from our lipidomics dataset, we noticed a clear separation on latent variable 1 (LV1) driven by lipids that correlated with disease status in African American or White groups (though the classification error for the cross-validation model was 0.5) (Fig. 3A). This separation between classes was even less clear when classifying racial groups independently by diabetes status (Suppl. Fig S7A and S7B). By comparing Dx-White from Dx-AA, we observed that the top 10 lipids that correlated positively with the presentation of diabetes in White individuals were long and very long chain TG in addition to monounsaturated species of phospholipids (Fig. 3B). Despite the classification model having high error, the lipid species identified using this model were significantly increased in Dx-White in comparison to NoDx-White and to Dx-AA (Fig. 3B). Additionally, some of the lipids reported to be markers of dyslipidemia, like ceramides and sphingosine, correlated positively with diabetes in White (both lipids) and African American (ceramides only) individuals (Suppl. Fig S7C and S7D). Our findings suggest that lipid profiles characterize the presentation of diabetes in White individuals, but not in African American individuals in this HANDLS subcohort.

Next, we analyzed inflammatory profiles using the same methodology. We observed a significant separation between Dx-AA and Dx-White (Fig. 3C), different from what was observed when selecting inflammatory features within each racial group (Suppl. Fig S8A and S8B). MCP-1, eotaxin, and IL-27 were important for the classification of diabetes in White individuals. Importantly, we saw that TNF-α, IL-6, and IL-1β (cytokines known to induce CRP) were also important for classifying diabetes in White individuals. In contrast, IL-17A, IL-1E, IL-17F, G-CSF, and IFN-γ (Th17-associated cytokines) were important contributors to the classification of diabetes in African Americans (Fig. 3D).

We also found that features characteristic of diabetes in White or African American groups were positively correlated to disease when compared with non-disease controls within each race (Suppl. Fig S8C and S8D). Taken together, our results identified markers of Th17-type inflammation associated with the presentation of diabetes in African Americans. We conclude that plasma cytokines generally reported to characterize diabetes (TNF-α, IL-6 and IL-1β) mainly do so in the White subcohort from the HANDLS study, but not in the African American subcohort.

### Relationships Between Lipids and Inflammatory Markers Exhibit Inverse Correlations with Clinical Measures of Diabetes in White and African American Participants Within the HANDLS Subcohort

We next investigated the relationship between lipids and inflammatory cytokines with respect to diabetes status. First, we calculated all possible permutations of ratios between all 128 lipids and 47 cytokines measured per subject to generate lipid:cytokine ratios. Then, we correlated each ratio with HbA1C or HOMA-IR, both significantly different between NoDx and Dx in both racial groups (Supp. Figure 2A and Suppl. Fig S9). We observed a striking inverse pattern in the relationships that correlated to diabetes in the White group compared to those in the African American cohort. The majority of lipid:cytokine ratios that significantly correlated with HbA1C (Fig. 3E) and HOMA-IR (Suppl. Figure 10) in White individuals were not significantly correlated in the African American group, and vice versa. Only a handful of ratios correlated significantly to clinical markers of diabetes in both groups; however, such correlations were weak. In the White cohort, most of the ratios that correlated positively to HbA1C (Fig. 3E) included at least one cytokine (MCP-1 or eotaxin) that was significantly increased in the White cohort in our previous analysis (Fig. 2 and 3). Conversely, in the African American group, relationships that included either MCP-1 and eotaxin correlated negatively to HOMA-IR (Suppl. Figure 10). In African Americans, we found that the inflammatory markers soluble CD40 ligand (sCD40L) and RANTES were present in most of the relationships positively correlated to HOMA-IR (Suppl. Figure 10). We conclude that lipid:cytokine relationships are inversely correlated to clinical markers of diabetes and insulin resistance in the White vs African American HANDLS cohort.

### Lipid and Inflammatory Features of Diabetes Seen in the HANDLS Subcohort Are Validated in a T2D Subcohort from the Multi-site AllofUs Diverse Study

The HANDLS subcohort is limited in size. Therefore, we used a second study to validate whether our main findings describing dramatic differences in lipids and inflammatory markers in the HANDLS subcohort would translate to a large well-powered cohort with high variability. Specifically, we investigated differences in clinical parameters associated with diabetes, dyslipidemia, and inflammation in a T2D cohort from the multi-ethnic study AllofUs (*N* = 17,339).

**Fig. 4:**
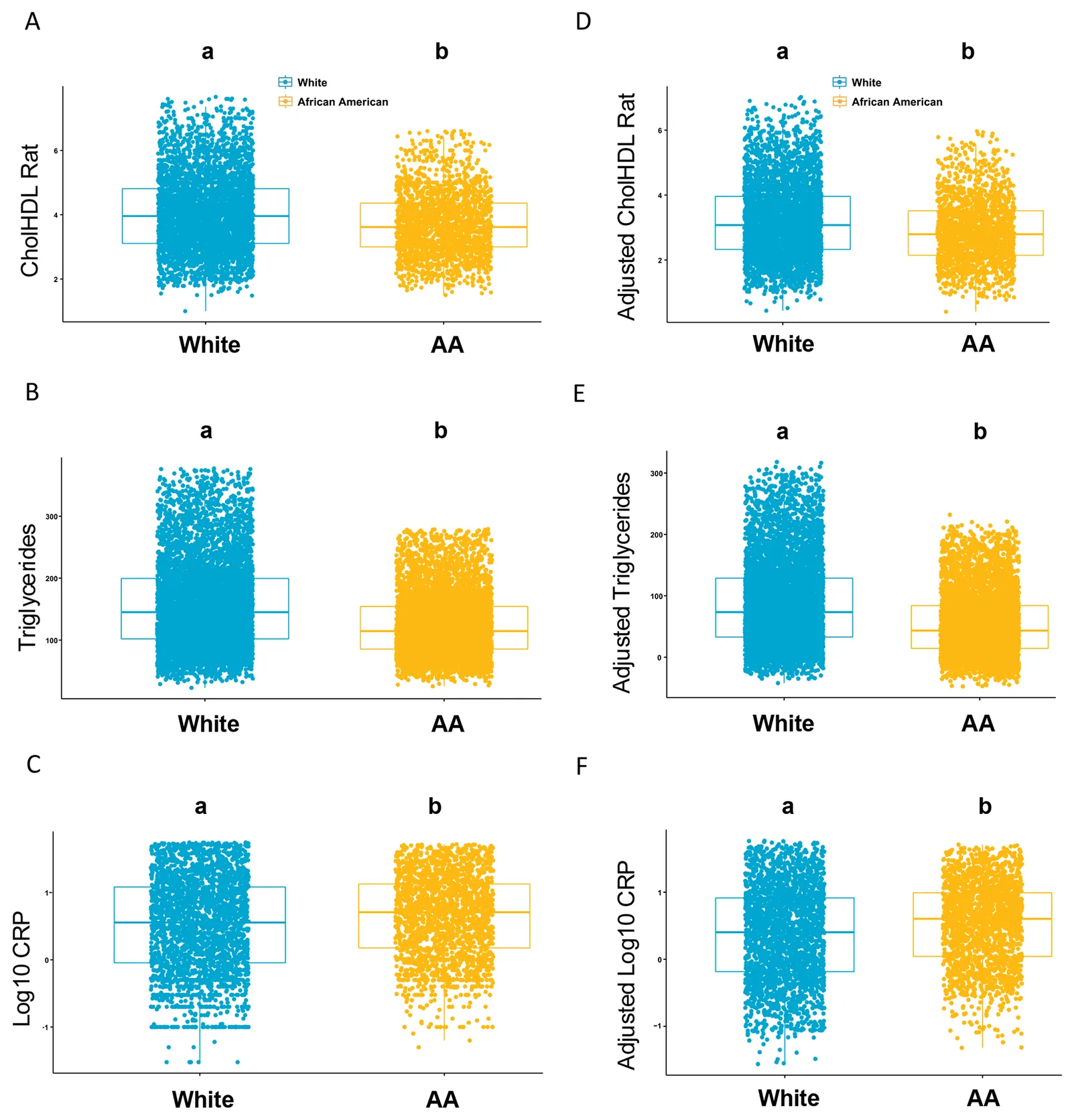
Clinical lipid and inflammatory parameters in AllofUs T2D subcohort confirm differential features seen in HANDLS diabetes subcohort. Clinical parameters that were differentially associated in White and AA groups from HANDLS subcohort were assessed using the multi-study AllofUs. Differences between the means of White and AA are plotted for CholHDLRat (**A**), triglycerides (**C**), and CRP (represented by logarithmic values due to exponential distribution) (**E**). A linear regression model was performed in the AllofUs T2D subcohort adjusting for variables body mass index (BMI) and age, biological variables used to match comparison groups in HANDLS diabetes subcohort. Adjusted differences between the means of Whites and AA are shown for CholHDLRat (**B**), triglycerides (**D**), and CRP (represented by logarithmic values due to exponential distribution) (**F**). The X axis represents race and the Y axis represents the clinical parameters evaluated. The White population is color represented in teal and the AA population is color represented in mustard. Statistical test used for comparison was Student T-test

By evaluating the same clinical measurements that were significantly modulated in the HANDLS diabetes subcohort in the AllofUs T2D subcohort, we noted similarities and differences (Fig. 4). We observed that only HbA1C, but not glucose and insulin, were significantly increased in African Americans with T2D compared to the White group when adjusted for BMI and age (Suppl. Fig. S11). Similar to the HANDLS subcohort, CholHDLRat (Fig. 4A) and total triglycerides (Fig. 4B) were significantly increased in the White population with T2D compared to African Americans with T2D. Opposite to the hs-CRP findings in the HANDLS subcohort, standard CRP levels were significantly increased in AA with T2D compared to White individuals with T2D (Fig. 4C). These findings remained even after adjusting for BMI and age (Fig. 4D–F). Due to limited available variables in the AllofUs dataset, we were unable to adjust for sex and poverty status in our analyses. Overall, the AllofUs T2D subcohort validated the findings from the HANDLS subcohort, confirming that dyslipidemia distinctively characterizes diabetes in White vs African American cohorts.

## Discussion

Our study demonstrates a disparity in the relationship of lipids and inflammatory mediators to indicators of glycemic control, potentially providing an explanation for how diabetes persists as a health disparity. In the HANDLS cohort, we demonstrated that triglycerides and a classic systemic inflammatory signature distinctively characterize diabetes in the White group but fail to characterize diabetes in African Americans. Conversely, diabetes in the African American cohort is characterized by a Th17-type inflammation. We validated elevated dyslipidemia in diabetes in White vs African American HANDLS participants in a large cohort of White and African American people with T2D through the AllofUs study.

Our results are consistent with the report indicating that minority groups, except for African Americans, are generally more likely to have high TGs and low HDL levels compared to White groups [45]. Results from our lipidomics analysis revealed that a variety of TGs were significantly increased in diabetes in our White cohort compared to the African American cohort which recapitulates previous conclusions from cohorts of European ancestry [46]. In concordance with our findings, studies in African Americans continuously report healthier lipid profiles [26, 45, 47, 48]. These findings support that plasma and clinical lipids are not uniformly related to diabetes risk and disease presentation, thereby contributing to the health disparity in disease burden in African Americans. Further, our data may explain why a racial disparity in the efficacy of lipid-lowering drugs to improve HbA1c persists [49], despite lipid-lowering drugs being equally, if not more, effective for cardiovascular risk in AA populations compared to White populations [50]. This suggests that the underlying metabolic pathways linking lipids to glycemic control differ between racial groups in this study through unknown mechanisms.

Our findings suggest that the classical marker of inflammation CRP mainly discriminates diabetes from non-diabetes cases in the White HANDLS cohort, but not in the African American group. Our findings in the White cohort are consistent with increased levels of CRP, IL-6, and TNF-α observed in T2D in several studies. In this study, Th17 cytokines were associated with diabetes in African Americans, consistent with studies demonstrating the importance of Th17 inflammation in T2D [24, 25, 50]. Our conclusions suggest that specific immune features reported in literature as relevant for diabetes could be impacted by the lack of diversity of the cohorts studied, hence contributing to the lack of efficacy in discovering and targeting immune pathways in T2D. The biological basis for these distinct inflammatory patterns may reflect fundamental differences in immune system activation between populations. Several studies support the Th17 mechanism we observed in African Americans, including the reported relationship of T2D-associated inflammation with Th17 cell cytokines [24, 25, 47] and the discovery that reduced IL-17 is associated with improved glucose management [50]. The Th17-type signature in AA was accompanied by higher levels of IL-33, and a role for IL-33 in modulating the balance between Th1/Th17 cells in autoimmune disorders has been postulated [51]. This suggests that diabetes-associated inflammation in African Americans may involve non-classical autoimmune-like mechanisms distinct from the metabolic inflammation typically described in predominantly White cohorts.

In this study, we observed race-dependent correlations of lipid-inflammatory marker ratios to HbA1c and HOMA-IR using an exploratory approach which strengthens discovery of novel biomarker relationships. Specifically, we uncovered broad correlations of lipids to RANTES and CD40L ratios in the AA cohort. Though its role and mechanisms remain under debate, the chemokine RANTES, also called CCL5, is associated with T2D, glucose intolerance, and obesity [52–54]. In a loss-of-function murine study, it was found that genetic deficiency of CD40L attenuated the development of diet-induced obesity, hepatic steatosis, and increased systemic insulin sensitivity [55]. Our findings correlating CD40L and RANTES to several types of lipids like PC, PE, cholesterol ester (CE), sphingomyelins (SM), and ceramides in Dx-AA could suggest the existence of an unexplored interplay among endogenous lipids, inflammation, diabetes, and insulin sensitivity. These data implicate differing mechanisms underlying insulin resistance, and these novel relationships require future validation studies.

When comparing clinical inflammatory markers, we found that hs-CRP findings from the HANDLS diabetes subcohort were not replicated when evaluating CRP values in the AllofUs T2D subcohort. This discrepancy may be attributed to technical differences between the two disparate assay methods in these studies. Although both hs-CRP and CRP measure the same molecule (C-reactive protein), the assays differ significantly in their detection ranges and lower limits of detection [56]. The lack of reproducibility between hs-CRP from the HANDLS subcohort and CRP values from the AllofUs subcohort could be due to technical variability when comparing measurements with different dynamic ranges: CRP values typically range from 10 to 1000 mg/L, while hs-CRP values range from 0.1 to 10 mg/L. However, a direct comparison using identical assay methods was not possible due to the unavailability of hs-CRP data in the AllofUs cohort, which limited our analysis to CRP measurements only.

Other factors could also account for the difference seen in CRP. Like mentioned above, in the AllofUs subcohort, data was not adjusted for sex and poverty status. It is known that the biological variable sex could have a potential impact on differences seen in diabetes presentation [57]. Likewise, literature directly implicates lower socioeconomic status in increased systemic inflammation and in increased risk of diabetes in AA [58–64], providing a mechanistic pathway through which social determinants of health could influence biological markers of disease [61, 64, 65]. Our data reaffirms the importance of including more sociobiological measurements in studies evaluating health and disease in diverse populations.

Comparing the clinical markers glucose, insulin, and CRP among our HANDLS subcohort, the AllofUs cohort (filtered by the range of HANDLS age and BMI, and self-reported race and diabetes status), and published literature gives context to our data. Among participants with diabetes, glucose levels were comparable between cohorts, with AAs showing mean values of 158.6 mg/dL (HANDLS) versus ~165 mg/ dL (AllofUs), and White participants showing 159.7 mg/ dL (HANDLS) compared to ~155 mg/dL (AllofUs). However, insulin levels differed substantially, with AllofUs participants demonstrating higher mean insulin concentrations (AA with diabetes, 20.6 μU/mL in HANDLS compared to ~35 μU/mL in AllofUs and White with diabetes, 14.4 μU/mL compared to ~25 μU/mL in AllofUs). For CRP, AAs with diabetes had comparable levels (3.06 mg/L in HANDLS compared to ~3.16 mg/L in AllofUs). As noted, CRP levels also varied between cohorts in the White population. White participants with diabetes demonstrated notably higher CRP in HANDLS (14.24 mg/L) compared to AllofUs (~1.58 mg/L). Among non-diabetic participants, both glucose and insulin levels were similar between cohorts. These findings align with established literature from large-scale diabetes studies. The Diabetes Prevention Program (DPP) reported baseline glucose levels of approximately 106 mg/dL and insulin levels of ~15 μU/mL among participants with impaired glucose tolerance [66], which are intermediate between our diabetic and non-diabetic groups. Similarly, the Multi-Ethnic Study of Atherosclerosis (MESA) found mean CRP levels of 3.1 mg/L among African Americans and 2.1 mg/L among White participants [67], consistent with our observed trend in increased CRP at baseline in AA populations and consistent with other literature [68]. The Jackson Heart Study, focusing on African Americans, reported mean glucose levels of 108 mg/dL and CRP levels of 4.2 mg/L [69], which approximates the average of the HANDLS diabetic and non-diabetic AA CRP values. The observed racial differences in insulin sensitivity and inflammatory markers are consistent with previous multi-ethnic studies, including the Insulin Resistance Atherosclerosis Study (IRAS), which demonstrated higher insulin levels and greater insulin resistance among African Americans compared to Whites across diabetes status categories [70]. These comparisons suggest that both our HANDLS and AllofUs cohorts exhibit clinical marker patterns consistent with established population-based studies, providing confidence in the generalizability of our core findings.

Our study includes several limitations. In the HANDLS subcohort, the limited sample size (N = 40), the absence of clinical information to accurately characterize T2D status (i.e.duration of condition and other blood parameters, etc.), the absence of information regarding anti-diabetic medications (i.e.treatment, time of treatment, etc.), and the absence of control for other relevant nutritional variables that can affect lipid and inflammatory profiles (i.e.food security, nutrition absorption, etc.). In the AllOfUs study, we were limited by the inability to use the same variables as HANDLS to match comparison groups, especially hs-CRP and poverty status. For the measurement of glycemic markers, we were limited to HbA1C and HOMA-IR. Though useful clinically, they are inherently limited because they are proxy measurements as opposed to oral glucose tolerance tests and an insulin sensitivity index, which are direct indicators.

In summary, we show that presentation of diabetes is metabolically and immunologically heterogeneous across populations and raise fundamental questions regarding how diabetes is managed in the clinic based on TG levels and CRP status. These mechanistic insights suggest that future research addressing the efficacy of Th17 anti-inflammatory therapy, especially in patients who do not achieve glycemic control targets through traditional approaches, is warranted. Finally, our study highlights the need for large-scale diabetes trials to be diverse to capture the full spectrum of disease presentation and intervention outcomes, thus paving the way for mechanistic understanding and individualized approaches to diabetes management.

## Supplementary Material

Supplementary Material

The online version contains supplementary material available at https://doi.org/10.1007/s40615-025-02642-z.

## Figures and Tables

**Table 1:** HANDLS subcohort demographics. A subcohort of 40 individuals from the HANDLS study was divided into 4 comparison groups based on disease status and race: White without diabetes (NoDx-White), White with diabetes (Dx-White), African Americans without diabetes (NoDx-AA), and African Americans with diabetes (Dx-AA). Groups were also equally distributed based on sex. Main variables used for control of group distribution were body mass index (BMI), age, and poverty status. Variable used as a potential surrogate for diabetes type determination was insulin use.

	NoDx-White (*N* = 10)	Dx-White (*N* = 10)	NoDx-AA (*N* = 10)	Dx-AA (*N* = 10)	Overall (*N* = 40)
**Sex**	5 (50%)	5 (50%)	5 (50%)	5 (50%)	20 (50%)
- Women	5 (50%)	5 (50%)	5 (50%)	5 (50%)	20 (50%)
- Men					
**Body mass index (BMI)**	29.46 (3.75)	32.54 (5.99)	30.03 (5.67)	29.03 (3.58)	30.27 (4.88)
Mean (SD)	30.47	33.86	27.64	29.35	29.76
Median	[22.66, 34.29]	[24.78, 41.28]	[23.18, 40.71]	[20.57, 34.01]	[20.57, 41.28]
[Min, Max]					
**BMI category**	4 (40%)	4 (40%)	6 (60%)	6 (60%)	20 (50%)
[20, 30]	6 (60%)	6 (60%)	4 (40%)	4 (40%)	20 (50%)
[30, 42]					
**Age**	51.35 (11.31)	49.53 (8.53)	52.3 (11.13)	54.56 (10.47)	51.93 (10.18)
Mean (SD)	55.6	52.1	57.45	58.25 [37.4,64.8]	55.45
Median	[32.5,64.7]	[34.4,58.1]	[30, 61.9]		[30.0, 64.8]
[Min, Max]					
**Poverty status**	9 (90%)	7 (70%)	9 (90%)	7 (70%)	32 (80%)
Above	1 (10%)	3 (30%)	1 (10%)	3 (30%)	8 (20%)
Below					
**Insulin use**	0 (0%)	3 (30%)	0 (0%)	1 (10%)	4 (10%)
Yes	10 (100%)	7 (70%)	10 (100%)	9 (90%)	36 (90%)
No					

## Data Availability

Not applicable.

## References

[1] Centers for Disease Control and Prevention (CDC). National Diabetes Statistics Report, 2024 [Internet]. Atlanta, GA; 2024 May [cited 2024 Jun 17]. Available from: https://www.cdc.gov/diabetes/php/data-research/?CDC_AAref_Val=https://www.cdc.gov/diabetes/data/statistics-report/index.html. Accessed 18 July 2025.

[2] Rodríguez JE, Campbell KM. Racial and ethnic disparities in prevalence and care of patients with type 2 diabetes. Clin Diabetes. 2017;35(1):66–70. 10.2337/cd15-0048 PMC5241767 28144049

[3] Haw JS, Shah M, Turbow S, Egeolu M, Umpierrez G. Diabetes complications in racial and ethnic minority populations in the USA. Curr Diab Rep. 2021;21(1): 2. 10.1007/s11892-020-01369-x PMC7935471 33420878

[4] Gravlee CC. How race becomes biology: embodiment of social inequality. Am J Phys Anthropol. 2009;139(1):47–57. 10.1002/ajpa.20983 19226645

[5] Bailey ZD, Krieger N, Agénor M, Graves J, Linos N, Bassett MT. Structural racism and health inequities in the USA: evidence and interventions. Lancet. 2017;389(10077):1453–63. 10.1016/S0140-6736(17)30569-X 28402827

[6] Good to Know. Race and type 2 diabetes. Clin Diabetes. 2020;38(4):403–4. 10.2337/cd20-pe04. PMC7566924 33132511

[7] Mahajan A, Taliun D, Thurner M, Robertson NR, Torres JM, Rayner NW, Fine-mapping type 2 diabetes loci to single-variant resolution using high-density imputation and islet-specific epigenome maps. Nat Genet. 2018;50(11):1505–13. 10.1038/s41588-018-0241-6 PMC6287706 30297969

[8] Vujkovic M, Keaton JM, Lynch JA, Miller DR, Zhou J, Tcheandjieu C, Discovery of 318 new risk loci for type 2 diabetes and related vascular outcomes among 1.4 million participants in a multi-ancestry meta-analysis. Nat Genet. 2020;52(7):680–91. 10.1038/s41588-020-0637-y PMC7343592 32541925

[9] Suzuki K, Hatzikotoulas K, Southam L, Taylor HJ, Yin X, Lorenz KM, Genetic drivers of heterogeneity in type 2 diabetes pathophysiology. Nature. 2024;627(8003):347–57. 10.1038/s41586-024-07019-6 PMC10937372 38374256

[10] Christensen DL, Witte DR, Kaduka L, Jørgensen ME, Borch-Johnsen K, Mohan V, Moving to an A1C-based diagnosis of diabetes has a different impact on prevalence in different ethnic groups. Diabetes Care. 2010;33(3):580–2. 10.2337/dc09-1843 PMC2827511 20009099

[11] Khalili D, Khayamzadeh M, Kohansal K, Ahanchi NS, Hasheminia M, Hadaegh F, Are HOMA-IR and HOMA-B good predictors for diabetes and pre-diabetes subtypes? BMC Endocr Disord. 2023;23(1):39. 10.1186/s12902-023-01291-9 PMC9926772 36788521

[12] Khan HA, Sobki SH, Ekhzaimy A, Khan I, Almusawi MA. Biomarker potential of C-peptide for screening of insulin resistance in diabetic and non-diabetic individuals. Saudi J Biol Sci. 2018;25(8):1729–32. 10.1016/j.sjbs.2018.05.027 PMC6303159 30591792

[13] Management of Hyperglycemia in Type 2 Diabetes, 2022. A consensus report by the American Diabetes Association (ADA) and the European Association for the Study of Diabetes (EASD) | Diabetes Care | American Diabetes Association [Internet]. [cited 2025 Jul 25]. Available from: https://diabetesjournals.org/care/article/45/11/2753/147671/Management-of-Hyperglycemia-in-Type-2-Diabetes 10.2337/dci22-0034 PMC10008140 36148880

[14] ElSayed NA, Aleppo G, Aroda VR, Bannuru RR, Brown FM, Bruemmer D 6. Glycemic targets: standards of care in diabetes—2023. Diabetes Care. 2022;46(Supplement_1):S97–110. 10.2337/dc23-S006 PMC9810469 36507646

[15] Matthews DR, Hosker JP, Rudenski AS, Naylor BA, Treacher DF, Turner RC. Homeostasis model assessment: insulin resistance and beta-cell function from fasting plasma glucose and insulin concentrations in man. Diabetologia. 1985;28(7):412–9. 10.1007/BF00280883 3899825

[16] Galicia-Garcia U, Benito-Vicente A, Jebari S, Larrea-Sebal A, Siddiqi H, Uribe KB, Pathophysiology of type 2 diabetes mellitus. IJMS. 2020;21(17):6275. 10.3390/ijms21176275 PMC7503727 32872570

[17] Calle MC, Fernandez ML. Inflammation and type 2 diabetes. Diabetes Metab. 2012;38(3):183–91. 10.1016/j.diabet.2011.11.006 22252015

[18] Mooradian AD. Dyslipidemia in type 2 diabetes mellitus. Nat Clin Pract Endocrinol Metab. 2009;5(3):150–9. 10.1038/ncpendmet1066 19229235

[19] Yang J, Wang M, Yang D, Yan H, Wang Z, Yan D, Integrated lipids biomarker of the prediabetes and type 2 diabetes mellitus Chinese patients. Front Endocrinol. 2023J;20(13):1065665. 10.3389/fendo.2022.1065665 PMC9897314 36743922

[20] Popko K, Gorska E, Stelmaszczyk-Emmel A, Plywaczewski R, Stoklosa A, Gorecka D, Proinflammatory cytokines IL-6 and TNF-α and the development of inflammation in obese subjects. Eur J Med Res. 2010;15(S2):120. 10.1186/2047-783X-15-S2-120 PMC4360270 21147638

[21] Stanimirovic J, Radovanovic J, Banjac K, Obradovic M, Essack M, Zafirovic S, Role of C-reactive protein in diabetic inflammation. Mediators Inflamm. 2022;3706508. 10.1155/2022/3706508. PMC9129992 35620114

[22] Swaroop J, Naidu J, Rajarajeswari D. Association of TNF-α with insulin resistance in type 2 diabetes mellitus. Indian J Med Res. 2012;135(1):127. 10.4103/0971-5916.93435 PMC3307173 22382194

[23] Ip B, Cilfone N, Zhu M, Kuchibhatla R, Azer M, McDonnell M, An inflammatory T cell signature predicts obesity-associated type 2 diabetes (HUM3P.262). J Immunol. 2015;194(1_Supplement):121.22–121.22.

[24] Ip B, Cilfone NA, Belkina AC, DeFuria J, Jagannathan-Bogdan M, Zhu M, Th17 cytokines differentiate obesity from obesity-associated type 2 diabetes and promote TNFα production. Obesity. 2016;24(1):102–12. 10.1002/oby.21243 PMC4688084 26576827

[25] Nicholas DA, Proctor EA, Agrawal M, Belkina AC, Van Nostrand SC, Panneerseelan-Bharath L, Fatty acid metabolites combine with reduced β oxidation to activate Th17 inflammation in human type 2 diabetes. Cell Metab. 2019;30(3):447–461.e5. 10.1016/j.cmet.2019.07.004 PMC8506657 31378464

[26] Bentley AR, Rotimi CN. Interethnic differences in serum lipids and implications for cardiometabolic disease risk in African ancestry populations. gh. 2017;12(2):141. 10.1016/j.gheart.2017.01.011 PMC5582986 28528248

[27] Zakai NA, Minnier J, Safford MM, Koh I, Irvin MR, Fazio S, Race-dependent association of high-density lipoprotein cholesterol levels with incident coronary artery disease. J Am Coll Cardiol. 2022;80(22):2104–15. 10.1016/j.jacc.2022.09.027 36423994

[28] Evans MK, Lepkowski JM, Powe NR, LaVeist T, Kuczmarski MF, Zonderman AB. Healthy aging in neighborhoods of diversity across the life span (HANDLS): overcoming barriers to implementing a longitudinal, epidemiologic, urban study of health, race, and socioeconomic status. Ethn Dis. 2010;20(3):267–75. PMC3040595 20828101

[29] U.S. Department of Agriculture. Automated Multiple-Pass Method - AMPM [Internet]. Food Surveys Research Group: Beltsville, MD; [cited 2024 Sep 4]. Available from: https://www.ars.usda.gov/northeast-area/beltsville-md-bhnrc/beltsville-human-nutrition-research-center/food-surveys-research-group/docs/ampm-usda-automated-multiple-pass-method/

[30] Ringnér M What is principal component analysis? Nat Biotechnol. 2008;26(3):303–4. 10.1038/nbt0308-303 18327243

[31] Tibshirani R, Walther G, Hastie T. Estimating the number of clusters in a data set via the gap statistic. J R Stat Soc Series B Stat Methodol. 2001;63(2):411–23.

[32] Tapp HS, Kemsley EK. Notes on the practical utility of OPLS. TrAC Trends Anal Chem. 2009;28(11):1322–7.

[33] Trygg J, Wold S. Orthogonal projections to latent structures (O-PLS). J Chemom. 2002;16(3):119–28.

[34] Worley B, Powers R. Multivariate analysis in metabolomics. CMB. 2012;1(1):92–107. 10.2174/2213235X11301010092 PMC4465187 26078916

[35] Barroeta-Espar I, Weinstock LD, Perez-Nievas BG, Meltzer AC, Siao Tick Chong M, Amaral AC, Distinct cytokine profiles in human brains resilient to Alzheimer’s pathology. Neurobiol Dis. 2019;121:327–37. 10.1016/j.nbd.2018.10.009 PMC6437670 30336198

[36] Wood LB, Winslow AR, Proctor EA, McGuone D, Mordes DA, Frosch MP, Identification of neurotoxic cytokines by profiling Alzheimer’s disease tissues and neuron culture viability screening. Sci Rep. 2015;5(1):16622. 10.1038/srep16622 PMC4643219 26564777

[37] Suhre K Genetic associations with ratios between protein levels detect new pQTLs and reveal protein-protein interactions. Cell Genom. 2024;4(3): 100506. 10.1016/j.xgen.2024.100506 PMC10943581 38412862

[38] Safi A, Giunti E, Melikechi O, Xia W, Melikechi N. Identification of blood plasma protein ratios for distinguishing Alzheimer’s disease from healthy controls using machine learning. Heliyon. 2025;11(3): e42349. 10.1016/j.heliyon.2025.e42349 PMC11840181 39981365

[39] Ramirez AH, Sulieman L, Schlueter DJ, Halvorson A, Qian J, Ratsimbazafy F, The all of us research program: data quality, utility, and diversity. Patterns. 2022;3(8): 100570. 10.1016/j.patter.2022.100570 PMC9403360 36033590

[40] Blake GJ, Ridker PM. Inflammatory bio-markers and cardiovascular risk prediction. J Intern Med. 2002;252(4):283–94. 10.1046/j.1365-2796.2002.01019.x 12366601

[41] Kalaiselvan P, Yingchoncharoen P, Thongpiya J, Motes A, Nugent K. COVID-19 infections and inflammatory markers in patients hospitalized during the first year of the pandemic. J Prim Care Community Health. 2023;14: 21501319231206911. 10.1177/21501319231206911 PMC10590050 37864436

[42] Lee JE, Nguyen HQ, Fan VS. Inflammatory markers and fatigue in individuals with moderate to severe chronic obstructive pulmonary disease. Nurs Res. 2024;73(1):54–61. 10.1097/NNR.0000000000000695 PMC10751060 38064303

[43] Rattik S, Engelbertsen D, Wigren M, Ljungcrantz I, Östling G, Persson M, Elevated circulating effector memory T cells but similar levels of regulatory T cells in patients with type 2 diabetes mellitus and cardiovascular disease. Diab Vasc Dis Res. 2019;16(3):270–80. 10.1177/1479164118817942 30574794

[44] Tan T, Xiang Y, Deng C, Cao C, Ren Z, Huang G, Variable frequencies of peripheral T-lymphocyte subsets in the diabetes spectrum from type 1 diabetes through latent autoimmune diabetes in adults (LADA) to type 2 diabetes. Front Immunol. 2022;24(13):974864. 10.3389/fimmu.2022.974864 PMC9449581 36091068

[45] Frank ATH, Zhao B, Jose PO, Azar KMJ, Fortmann SP, Palaniappan LP. Racial/ethnic differences in dyslipidemia patterns. Circulation. 2014;129(5):570–9. 10.1161/CIRCULATIONAHA.113.005757 PMC4212818 24192801

[46] Fernandez C, Surma MA, Klose C, Gerl MJ, Ottosson F, Ericson U, Plasma lipidome and prediction of type 2 diabetes in the population-based Malmö diet and cancer cohort. Diabetes Care. 2020;43(2):366–73. 10.2337/dc19-1199 31818810

[47] Jagannathan-Bogdan M, McDonnell ME, Shin H, Rehman Q, Hasturk H, Apovian CM, Elevated proinflammatory cytokine production by a skewed T cell compartment requires monocytes and promotes inflammation in type 2 diabetes. J Immunol. 2011;186(2):1162–72. 10.4049/jimmunol.1002615 PMC3089774 21169542

[48] Mcintosh MS, Kumar V, Kalynych C, Lott M, Chang JL, Lerman RH. Racial differences in blood lipids lead to underestimation of cardiovascular risk in Black women in a nested observational study. Glob Adv Health Med. 2013;2(2):76–9. 10.7453/gahmj.2012.076 PMC3833531 24416666

[49] Cromer SJ, Thaweethai T, Wexler DJ. Racial/ethnic and socioeconomic disparities in achievement of treatment goals within a clinical trial: a secondary analysis of the ACCORD trial. Diabetologia. 2023;66(12):2261–74. 10.1007/s00125-023-05997-2 PMC10942722 37715820

[50] Sumarac-Dumanovic M, Jeremic D, Pantovic A, Janjetovic K, Stamenkovic-Pejkovic D, Cvijovic G, Therapeutic improvement of glucoregulation in newly diagnosed type 2 diabetes patients is associated with a reduction of IL-17 levels. Immunobiology. 2013;218(8):1113–8. 10.1016/j.imbio.2013.03.002 23623393

[51] Liu X, Xiao Y, Pan Y, Li H, Zheng SG, Su W. The role of the IL-33/ST2 axis in autoimmune disorders: Friend or foe? Cytokine & growth factor reviews [Internet]. 2019 Dec [cited 2025 Jul 27];50. Available from: https://pubmed.ncbi.nlm.nih.gov/31085085/10.1016/j.cytogfr.2019.04.004 31085085

[52] Chou SY, Ajoy R, Changou CA, Hsieh YT, Wang YK, Hoffer B. CCL5/RANTES contributes to hypothalamic insulin signaling for systemic insulin responsiveness through CCR5. Sci Rep. 2016;6(1):37659. 10.1038/srep37659 PMC5127185 27898058

[53] Dworacka M, Krzyżagórska E, Iskakova S, Bekmukhambetov Y, Urazayev O, Dworacki G. Increased circulating RANTES in type 2 diabetes. Eur Cytokine Netw. 2014;25(3):46–51. 10.1684/ecn.2014.0355 25373852

[54] Yao L, Herlea-Pana O, Heuser-Baker J, Chen Y, Barlic-Dicen J. Roles of the chemokine system in development of obesity, insulin resistance, and cardiovascular disease. J Immunol Res. 2014;2014:1–11. 10.1155/2014/181450 PMC3987870 24741577

[55] Poggi M, Engel D, Christ A, Beckers L, Wijnands E, Boon L, CD40L deficiency ameliorates adipose tissue inflammation and metabolic manifestations of obesity in mice. ATVB. 2011;31(10):2251–60. 10.1161/ATVBAHA.111.231357 21817098

[56] Wolska A, Remaley ATCRP, High-Sensitivity CRP. What’s in a name? The Journal of Applied Laboratory Medicine. 2022;7(6):1255–8. 10.1093/jalm/jfac076 36136105

[57] Cherian CM, Reeves HR, De Silva D, Tsao S, Marshall KE, Rideout EJ Consideration of sex as a biological variable in diabetes research across twenty years. Biol Sex Differ [Internet]. 2024 Feb 26 [cited 2025 Jul 15];15(1). Available from: https://bsd.biomedcentral.com/articles/ 10.1186/s13293-024-00595-2 PMC10895746 38409052

[58] Arnold NS The association between poverty and gene expression within peripheral blood mononuclear cells in a diverse Baltimore City cohort. PloS one [Internet]. 2020 Sep 24 [cited 2025 Jul 27];15(9). Available from: https://pubmed.ncbi.nlm.nih.gov/32970748/10.1371/journal.pone.0239654 PMC7514036 32970748

[59] Boylan JM, Cundiff JM, Fuller-Rowell TE, Ryff CD. Childhood socioeconomic status and inflammation: psychological moderators among Black and White Americans. Health psychology : Official journal of the Division of Health Psychology, American Psychological Association [Internet]. 2020 Jun [cited 2025 Jul 27];39(6). Available from: https://pubmed.ncbi.nlm.nih.gov/32212770/10.1037/hea0000866 PMC7437114 32212770

[60] Butler AM. Social determinants of health and racial/ethnic disparities in type 2 diabetes in youth. Curr Diabetes Rep [Internet]. 2017;17(8):60. [cited 2025 Jul 27]. Available from: https://pubmed.ncbi.nlm.nih.gov/28664253/. 10.1007/s11892-017-0885-0 PMC5728112 28664253

[61] Cooper ZW, Mowbray O, Johnson L. Social determinants of health and diabetes: using a nationally representative sample to determine which social determinant of health model best predicts diabetes risk. Clin Diabetes Endocrinol. 2024;10(1):4. 10.1186/s40842-023-00162-5 PMC10894485 38402223

[62] Gaskin DJ Disparities in diabetes: the nexus of race, poverty, and place. American journal of public health [Internet]. 2014 Nov [cited 2025 Jul 27];104(11). Available from: https://pubmed.ncbi.nlm.nih.gov/24228660/10.2105/AJPH.2013.301420 PMC4021012 24228660

[63] Muscatell KA, Brosso SN, Humphreys KL. Socioeconomic status and inflammation: a meta-analysis. Mol Psychiatry. 2020;25(9):2189–99. 10.1038/s41380-018-0259-2 PMC6814496 31628416

[64] Van Dyke ME, Vaccarino V, Dunbar SB, Pemu P, Gibbons GH, Quyyumi AA, Socioeconomic status discrimination and C-reactive protein in African-American and White adults. Psychoneuroendocrinology. 2017;82:9–16. 10.1016/j.psyneuen.2017.04.009 PMC5519320 28482209

[65] Williams DR, Priest N, Anderson NB. Understanding associations among race, socioeconomic status, and health: patterns and prospects. Health Psychol. 2016;35(4):407–11. 10.1037/hea0000242 PMC4817358 27018733

[66] Knowler AQ, Knowler WC, Barrett-Connor E, Fowler SE, Hamman RF, Lachin JM, Reduction in the incidence of type 2 diabetes with lifestyle intervention or metformin. N Engl J Med. 2002;346(6):393–403. 10.1056/NEJMoa012512 PMC1370926 11832527

[67] Cushman M, Arnold AM, Psaty BM, Manolio TA, Kuller LH, Burke GL, C-reactive protein and the 10-year incidence of coronary heart disease in older men and women: the cardiovascular health study. Circulation. 2005;112(1):25–31 Jul 5; 10.1161/CIRCULATIONAHA.104.504159 15983251

[68] Farmer HR, Wray LA, Xian Y, Xu H, Pagidipati N, Peterson ED, Racial differences in elevated C-reactive protein among US older adults. J Am Geriatr Soc. 2020;68(2):362–9. 10.1111/jgs.16187 PMC8211020 31633808

[69] Carnethon MR, Loria CM, Hill JO, Sidney S, Savage PJ, Liu K, Risk factors for the metabolic syndrome: the Coronary Artery Risk Development in Young Adults (CARDIA) study, 1985–2001. Diabetes Care. 2004;27(11):2707–15. 10.2337/diacare.27.11.2707 15505009

[70] Haffner SM, Howard G, Mayer E, Bergman RN, Savage PJ, Rewers M, Insulin sensitivity and acute insulin response in African-Americans, non-Hispanic whites, and Hispanics with NIDDM: the insulin resistance atherosclerosis study. Diabetes. 1997;46(1):63–9. 10.2337/diab.46.1.63 8971083

