# Supplementary material for: Comparative Analysis of White and African American Groups Reveals Unique Lipid and Inflammatory Features of Diabetes
Source: J Racial Ethn Health Disparities. 2025 Sep 26;13(6):5008–24. doi: 10.1007/s40615-025-02642-z (PMC13097115; doi:10.1007/s40615-025-02642-z)
Supplement: Supplementary file 1 [file 40615_2025_2642_MOESM1_ESM.docx]

**Comparative analysis of White and African American groups reveals unique lipid and inflammatory features of diabetes**

Gabriela Pacheco Sanchez*^1^, Miranda Lopez*^1,2^, Leandro M. Velez^2^, Ian Tamburini^2^, Naveena Ujagar^1^, Julio Ayala Angulo^1^, Gabriela De Robles^1,2^, Hannah Choi^1^, John Arriola^1^, Rubina Kapadia^1^, Alan B. Zonderman^3^, Michele K. Evans^3^, Cholsoon Jang^2^, Marcus M. Seldin^2^, Dequina A. Nicholas^1,2^

1 Department of Molecular Biology and Biochemistry, School of Biological Sciences, University of California, Irvine. California, USA

2 Department of Biological Chemistry, and Center for Epigenetics and Metabolism, School of Medicine, University of California, Irvine. California, USA

3 The Laboratory of Epidemiology and Population Sciences, National Institute on Aging, National Institutes of Health. Maryland, USA.

*These authors contributed equally

Corresponding author: Dequina Nicholas - [dequinaa@uci.edu](mailto:dequinaa@uci.edu), <https://orcid.org/0000-0003-4996-2190>

Disclosure Statement: The authors have nothing to disclose.

**SUPPLEMENTARY FIGURES:**

**Supplemental Figure S1. Study experimental design using the diverse HANDLS and AllOfUs studies. A)** HANDLS subcohort schematic showing equal distribution of participants by race and sex. Participants were also equally divided by diabetes status. Techniques employed for the generation of datasets are shown in B, C, and D. Assessment of dietary intake data and immune phenotyping was also performed but measurements were not statistically significant. **B)** Clinical parameters measured in serum consisted of glucose measurements (HbA1C and glucose), lipids measurements (cholesterol, triglycerides, HDL, LDL, and VLDL), and inflammation measurements (C-reactive protein). **C)** Plasma lipidomes profiles were generated using targeted lipidomics. **D)** Plasma cytokines profiles were generated using multiplex Luminex platform. All independent analysis consisted of statistical and bioinformatic assessment and visualization tools. **E)** Integrative analysis of HANDLS subcohort datasets generated was performed. Lastly, **F)** a group of individuals from the AllOfUs study were subset to create a Type 2 Diabetes cohort including African American and White groups. Due to availability of comparable clinical data, variables triglycerides, HbA1C, CRP, insulin and glucose were evaluated.

A

**White	AA**

**White	AA**

**Supplemental Figure S2. Statistical post-ANOVA comparison of insulin.** Bar/dot graph showing results from multiple statistical comparisons of insulin levels among Whites and AA without and with diabetes. Insulin levels for all participants **(A)** and adjusted for participants who were not prescribed insulin **(B)** are shown . Blue = People without diabetes, red = people with diabetes. Statistical analysis performed using Two-way ANOVA with followed by Fisher’s LSD post-comparison test (unadjusted p-values). Statistical post-ANOVA comparisons were performed only between matching groups based on diabetes status and race and comparisons between groups with and without diabetes were excluded from the analysis. P-values obtained from analysis were represented using a statistical letter system, where significantly different p-values are represented by different letters and non-significant p-values are represented by same letters.

A

B

**Supplemental Figure S3. Statistical post-ANOVA comparison of HbA1C and Glucose.** Bar/dot graph showing results from multiple post-anova comparison of **(A)** HbA1C and **(B)** fasting glucose among white and AA with and without diabetes. Statistical analysis performed using Two-way ANOVA with followed by Fisher’s LSD post-comparison test (unadjusted p-values). Statistical post-ANOVA comparisons were performed only between matching groups based on diabetes status and race and comparisons between groups with and without diabetes were excluded from the analysis. P-values obtained from analysis were represented using a statistical letter system, where significantly different p-values are represented by different letters and non-significant p-values are represented by same letters.

**White	AA**

**White	AA**

A

B

**Supplemental Figure S4. Statistical post-ANOVA comparison of Cholesterol/HDL ratio (CholHDLRat) and high sensitivity C-reactive protein (hsCRP) adjusting for statins (lipid lowering drug) use.** Bar/dot graph showing results from multiple statistical comparison of CholHDLRat **(A)** and hsCRP **(B)** among Whites and AA without and with diabetes. Blue = People without diabetes, red = people with diabetes. Statistical analysis performed using Two-way ANOVA with followed by Fisher’s LSD post-comparison test (unadjusted p-values). Statistical post-ANOVA comparisons were performed only between matching groups based on diabetes status and race and comparisons between groups with and without diabetes were excluded from the analysis. P-values obtained from analysis were represented using a statistical letter system, where significantly different p-values are represented by different letters and non-significant p-values are represented by same letters.

**White	AA**

**White	AA**

**White	AA**

**IL-6	TNF-a	IL-1b**

**Supplemental Figure S5. Statistical post-ANOVA comparison of classical inflammatory markers IL-6, TNF-a, and IL-1b in HANDLS subcohort.** Bar/dot graph showing results from post-ANOVA multiple statistical comparisons among Whites and AA without and with diabetes for IL-6 (left), TNF-a (middle), and IL-1b (right). Blue = People without diabetes, red = people with diabetes. Statistical analysis was performed using Two-way ANOVA with followed by Fisher’s LSD post-comparison test (unadjusted p-values). Statistical post-ANOVA comparisons were performed only between matching groups based on diabetes status and race and comparisons between groups with and without diabetes were excluded from the analysis. P-values obtained from analysis were represented using a statistical letter system, where significantly different p-values are represented by different letters, and non-significant p-values are represented by the same letters.

C

A

B

**Supplemental Figure S6. Gating strategy for CD4+ Central Memory T cells.**

**A)** First, cells were gated by time of sample acquisition followed by doublet discrimination using FCS-H and FSC-A. Next live cells were gated based on viability dye. Then for size and granularity for lymphocytes and myeloid cells based on SSC-A and FSC-A. After that CD3+ T cells were gated based on CD3 and CD19 antibodies. From CD3+ T cells, CD4+ T cells were gated based on antibodies CD4 and CD8. Next, CD4+CD45RO+ T cells were gated based on CD45RO and CD45RA antibodies. **B)** Lastly, CD4+ Central memory T cells were gated based on markers CCR7 and CD62L. **C)** Stain controls were included in the experiment to verify positive populations. Percentages on plot represent frequencies based on parent population.

A

D

C

B

**NoDx-White vs Dx-White**

**NoDx-AA vs Dx-AA**

**Scores on LV1 distinguishing NoDx-AA from Dx-AA**

**Scores on LV1 distinguishing NoDx-White from Dx-White**

**Supplemental Figure S7. Lipid features characteristics of White and AA with diabetes in a diverse HANDLS subcohort.**

Orthogonal partial least squares discriminant analysis (OPLS-DA) plot of lipids features correlated with presentation of diabetes in White **(A)** and AA **(C)**. Blue dots refer to group without diabetes and red dots refer to group with diabetes. X-axis represents scores on latent variable (LV) 1 and Y-axis represent scores on LV2 not used for analysis. Bar graph plot displaying scores on LV1 showing lipids that distinguish diabetes in White **(B)** and AA **(D)**. Top 10 features that correlated positively (red) and negatively (blue) to diabetes in White and AA are colored in the charts.

A

D

C

B

**Scores on LV1 distinguishing NoDx-AA from Dx-AA**

**Scores on LV1 distinguishing NoDx-White from Dx-White**

**NoDx-AA vs Dx-AA**

**Dx-White vs NoDx-White**

**Supplemental Figure S8. Inflammatory features characteristics of White and AA with diabetes in a diverse HANDLS subcohort.**

Orthogonal partial least squares discriminant analysis (OPLS-DA) plot of lipids features correlated with presentation of diabetes in White **(A)** and AA **(C)**. Blue dots refer to participants without diabetes and red dots refer to participants with diabetes. X-axis represents scores on latent variable (LV) 1 and Y-axis represents scores on LV2 not used for analysis. Bar graph plot displaying scores on LV1 showing lipids that distinguish diabetes in white **(B)** and AA **(D)**. Top 10 features that correlated positively (red) and negatively (blue) to diabetes in white and AA are colored in the charts.

**Supplemental Figure S9. Statistical post-ANOVA comparison of homeostasis model assessment for insulin resistance (HOMA-IR).** Bar/dot graph showing results from multiple statistical comparisons of HOMA-IR values among Whites and AA without and with diabetes. Blue = People without diabetes, red = people with diabetes. Statistical analysis performed using Two-way A NOVA with followed by Fisher’s LSD post-comparison test (unadjusted p-values). Statistical post-ANOVA comparisons were performed only between matching groups based on diabetes status and race and comparisons between groups with and without diabetes were excluded from the analysis. P-values obtained from analysis were represented using a statistical letter system, where significantly different p-values are represented by different letters and non-significant p-values are represented by same letters.

**White	AA**

**Pearson correlation coefficient of Lipids:Cytokines relations ratios with HOMA-IR**

**Lipids:Cytokines Ratio Correlations with HOMA-IR**

**Whites**

**African Americans**

**Supplemental Figure S10. Modulatory relationships of lipids and inflammatory markers in White and AA groups correlate inversely to HOMA-IR.** Volcano plots showing all permutated lipid:cytokine correlations with HOMA-IR in White and AA. Briefly, all possible ratios were calculated for each lipid:cytokine, correlated to HOMA-IR, and subset for all correlations that were significant in each racial group only or in both. Correlations uniquely significant in white are colored in blue and correlations uniquely significant in AA are colored in yellow . Significant correlations in both groups are represented by red triangles. X axis indicates the pearson correlation coefficient. Y axis indicates the –log10 of p-values for the lipid/cytokine ratio correlated to HOMA-IR. The dotted red line represents threshold of significance values, above p-value<0.05 and below p-value>0.05.

C

**White	AA**

**Glucose**

**a	a**

E

**a	b**

**White	AA**

**Insulin**

A

**White	AA**

**HbA1C**

**a	b**

B

**White	AA**

**Adjusted HbA1C**

**a	b**

D

**a	a**

**Adjusted Glucose**

**White	AA**

F

**Adjusted Insulin**

**White	AA**

**a	a**

**Supplemental Figure S11. Clinical lipid and inflammatory parameters in AllofUs T2D subcohort confirm differential features seen in HANDLS diabetes subcohort.** Clinical parameters that were differentially associated to diabetes in socially diverse populations from HANDLS subcohort were assessed using the multi-study AllofUs. Differences between the means of white and AA are plotted for HbA1C **(A)**, glucose **(C)**, and insulin **(E)**. A linear regression model was performed in the AllofUs T2D subcohort adjusting for variables body mass index (BMI) and age, biological variables used to match comparison groups in HANDLS diabetes subcohort. Adjusted differences between the means of whites and AA are shown for HbA1C **(B)**, glucose **(D)**, and insulin **(F)**. X axis represent race of group and Y axis represents clinical parameters evaluated. White population are colored represented in teal and AA population are colored represented in mustard. Statistical test used for comparison was student T-test.

**SUPPLEMENTARY TABLES:**

**Supplementary Table S1**. Flow cytometry panel used to phenotype cellular populations in PBMCs.

| **Marker** | **Fluorophore** | **Clone** | **Purpose** | **Supplier** | **Cat. #** | **Dilution** |
| --- | --- | --- | --- | --- | --- | --- |
| CCR7/ CD197 | PE/Fire 810 | G043H7 | Memory T cells | Biolegend | 353269 | 200 |
| CD11c | Pacific Blue | 3.9 | Dendritic cells | Biolegend | 301626 | 100 |
| CD14 | PE/Cyanine7 | 63D3 | Monocytes | Biolegend | 367112 | 100 |
| CD16 | BUV 805 | 3G8 | NKC & monocyte | BD Biosciences | 748850 | 400 |
| CD19 | AF647 | HIB19 | B cells | Biolegend | 302220 | 400 |
| CD25 | APC-R700 | 2A3 | Regulatory T cells | BD Biosciences | 565106 | 200 |
| CD3 | PerCP | OKT3 | T cells | Biolegend | 317338 | 200 |
| CD4 | Spark YG 593 | SK3 | helper T cells | Biolegend | 344672 | 100 |
| CD45 | BV 510 | 2D1 | Pan leukocytes | Biolegend | 368526 | 400 |
| CD45RA | BV 785 | HI100 | Naive T cells | Biolegend | 304140 | 400 |
| CD56 | BUV496 | NCAM16.2 | NK cells | BD Biosciences | 750479 | 50 |
| CD69 | BUV 563 | FN50 | T cell activation | BD Biosciences | 748764 | 100 |
| CD8 | Spark Blue 550 | SK1 | Cytotoxic T cells | Biolegend | 344760 | 200 |
| FOXP3 | PE-Cy5.5 | PCH101 | Treg | ThermoFisher Scientific | 35-4776-42 | 50 |
| HLA-DR | BV711 | L243 | Antigen presenting cells | Biolegend | 307644 | 100 |
| PD1 | Super Bright 645 | MIH4 | T cell activation | ThermoFisher Scientific | 64-9969-42 | 100 |
| TIGIT | BUV615 | 741182 | T cell exhaustion | BD Biosciences | 752314 | 100 |
| IgM | BUV395 | G20-127 | B cell | BD Biosciences | 563903 | 400 |
| IgD | BUV661 | IA6-2 | Mature B cells | BD Biosciences | 741637 | 400 |
| CD45RO | BUV737 | UCHL1 | Memory T cells | BD Biosciences | 748368 | 100 |
| CD62L | APC-Fire 810 | DREG-56 | T cell subsets | Biolegend | 304866 | 400 |
| CD44 | Nova Fluor Blue 610/70S | IM7 | T cells | ThermoFisher Scientific | M010T02B06 | 100 |
| Live/Dead | Zombie NIR | --- | Cell viability | Biolegend | 423106 | 3200 |
| Fc Receptor | Human TruStain FcX | --- | Block human FC receptors to prevent false positives or false negatives | Biolegend | 422302 | 50 |

**Supplementary Table S2.** List of dietary intake lipids and statistical differences (p-values) Using Two-way ANOVA and post-ANOVA tests comparing all four groups in the HANDLS subcohort (N=40)

**Table legend:** A subcohort of 40 individuals from the HANDLS study were divided into 4 comparison groups based on disease status and race: White without diabetes (NoDx-White), White with diabetes (Dx-White), African Americans without diabetes (NoDx-AA), and African Americans with diabetes (Dx-AA). Values were transformed using Box-Cox transformation and comparisons were made using the two-way Anova test with post-anova comparisons using the Fisher LSD’s test. P-values are displayed as decimal numbers.

| **Dietary Intake type of Food** | **ANOVA Overall** | **Race** | **Diabetes** | **Interaction of race x diabetes** | **NoDx-White**  **vs**  **Dx-White** | **NoDx-AA**  **vs**  **Dx-AA** | **Dx-White**  **vs**  **Dx-AA** | **NoDx-White**  **vs**  **NoDx-AA** |
| --- | --- | --- | --- | --- | --- | --- | --- | --- |
| Ifa4 | 0.4839 | 0.3267 | 0.2271 | 0.9484 | 0.4160 | 0.3663 | 0.5154 | 0.4586 |
| Ifa6 | 0.3427 | 0.2655 | 0.1794 | 0.5927 | 0.5611 | 0.1855 | 0.6783 | 0.2452 |
| Ifa8 | 0.5159 | 0.3589 | 0.2359 | 0.9343 | 0.3683 | 0.4326 | 0.4787 | 0.5532 |
| Ifa10 | 0.6516 | 0.5139 | 0.2897 | 0.8088 | 0.3574 | 0.5605 | 0.5272 | 0.7706 |
| Ifa12 | 0.6113 | 0.3092 | 0.5564 | 0.5206 | 0.3854 | 0.9692 | 0.2425 | 0.7882 |
| **Ifa14 *** | **0.0256** | **0.0072** | 0.2884 | 0.2887 | 0.9996 | 0.1364 | 0.2175 | **0.0086** |
| Ifa16 | 0.4393 | 0.6300 | 0.1855 | 0.4045 | 0.7224 | 0.1296 | 0.8018 | 0.3534 |
| Ifa16_1 | 0.4580 | 0.9928 | 0.5767 | 0.1350 | 0.4991 | 0.1477 | 0.2896 | 0.2840 |
| Ifa18 | 0.3228 | 0.4286 | 0.1309 | 0.4526 | 0.5816 | 0.1118 | 0.9769 | 0.2773 |
| Ifa18_1 | 0.6823 | 0.8135 | 0.3078 | 0.5396 | 0.7707 | 0.2499 | 0.5483 | 0.7888 |
| Ifa18_2 | 0.7162 | 0.7022 | 0.5078 | 0.3874 | 0.8850 | 0.2823 | 0.3787 | 0.7313 |
| Ifa18_3 | 0.7610 | 0.7125 | 0.4071 | 0.5705 | 0.8517 | 0.3249 | 0.8877 | 0.5087 |
| Ifa18_4 | 0.6983 | 0.8370 | 0.2598 | 0.7708 | 0.3159 | 0.5510 | 0.7253 | 0.9516 |
| Ifa20_1 | 0.4208 | 0.4525 | 0.2745 | 0.3055 | 0.9607 | 0.1373 | 0.2115 | 0.8442 |
| Ifa20_4 | 0.0946 | 0.7932 | 0.2647 | 0.0244 | 0.3951 | **0.0187** | 0.0728 | 0.1489 |
| Ifa20_5n3 | 0.6478 | 0.3145 | 0.6068 | 0.55444 | 0.4352 | 0.9565 | 0.2604 | 0.7664 |
| Ifa22_1 | 0.2885 | 0.6881 | 0.4275 | 0.0868 | 0.5024 | 0.0783 | 0.3441 | 0.1345 |
| Ifa22_5n3 | 0.1117 | **0.0188** | 0.5527 | 0.8809 | 0.5990 | 0.7530 | 0.0731 | 0.1112 |
| Ifa22_6n3 | 0.5814 | 0.2157 | 0.5645 | 0.8135 | 0.5660 | 0.8093 | 0.2965 | 0.4743 |
| MonoFat | 0.6792 | 0.8269 | 0.3225 | 0.4974 | 0.8236 | 0.2403 | 0.5259 | 0.7441 |
| PolyFat | 0.6928 | 0.7249 | 0.4988 | 0.3569 | 0.8606 | 0.2606 | 0.3685 | 0.6849 |
| Saturated Fat | 0.3251 | 0.2889 | 0.2182 | 0.3604 | 0.8186 | 0.1320 | 0.9161 | 0.1653 |
| Fat | 0.5599 | 0.4762 | 0.6840 | 0.1554 | 0.9196 | 0.1605 | 0.5873 | 0.4383 |
| Carbohydrates | 0.4358 | 0.4762 | 0.6840 | 0.1554 | 0.4666 | 0.1964 | 0.6084 | 0.1335 |
| Total sugar | 0.4229 | 0.3679 | 0.5996 | 0.1926 | 0.1973 | 0.5760 | 0.7703 | 0.1220 |
| Protein | 0.1265 | 0.5151 | 0.8180 | **0.0233** | 0.1394 | 0.0741 | 0.2340 | **0.0392** |
| Energy | 0.4654 | 0.5553 | 0.5393 | 0.1797 | 0.5998 | 0.1683 | 0.5880 | 0.1735 |
| Dash Saturated Fat | 0.5206 | 0.5399 | 0.2238 | 0.5399 | 0.1976 | 0.6643 | 0.3873 | >0.9999 |
| Dash Total Fat | 0.8178 | 0.3922 | 0.7271 | 0.8130 | 0.6790 | 0.9365 | 0.6598 | 0.4399 |

**Supplementary Table S3. List of 128 lipids evaluated using targeted lipidomics in HANDLS subcohort.**

**Table legend:** A subcohort of 40 individuals from the HANDLS study were divided into 4 comparison groups based on disease status and race: White without diabetes (NoDx-White), White with diabetes (Dx-White), African Americans without diabetes (NoDx-AA), and African Americans with diabetes (Dx-AA).

| **Class of lipid** | **Lipid Species** | |
| --- | --- | --- |
| Cholesterol Ester | 1. CE.16.1 2. CE.16.2 3. CE.18.0 4. CE.18.1 5. CE.20.0 | 6. CE.20.1 7. CE.20.5 8. CE.22.0 9. CE.22.5 10. CE.24.0 |
| Cearamides | 1. Cearamide.d18:1/14:0 2. Cearamide.d18:1/16:1 3. Cearamide.d18:1/18:0 4. Cearamide.d18:1/18:1 5. Cearamide.d18:1/18:3 6. Cearamide.d18:1/20:0 | 7. Cearamide.d18:1/22:0 8. Cearamide.d18:1/22:1 9. Cearamide.d18:1/24:0 10. Cearamide.d18:1/24:1 11. Cearamide.d18:1/26:1 12. Cearamide.d18:1/28:3 |
| Diacylglycerides | 1. DG.28:0 2. DG.30:0 3. DG.30:1 4. DG.32:0 5. DG.32:1 6. DG.32:2 7. DG.34:0 8. DG.34:1 | 9. DG.34:2 10. DG.34:3 11. DG.38:0 12. DG.40:0 13. DG.46:4 14. DG.48:10 15. DG.48:11 16. DG.48:12 |
| Glucosyl / Galactosyl Cearamide | 1. Glucosyl/Galactosyl Cearamide.d18:1/22:1 | 2. Glucosyl/Galactosyl Cearamide.d18:1/22:5 |
| Lyso - phosphatidilethanolamine | 1. LPE.16:0 |  |
| Monoglycerides | 1. MG.18:1 | 2. MG.20:5 |
| Phosphatidylcholine | 1. PC.32:0 2. PC.34:0 | 3. PC.34:1 4. PC.38:4 |
| Phosphatidylethanolamine | 1. PE.34:1 2. PE.36:1 3. PE.38:4 4. PE.44:11 | 5. PE.O-36:2 6. PE.O-36:3 7. PE.O-36:5 8. PE.O-38:5 9. PE.O-38:6 |
| Phosphatidylserine | 1. PS.34:0 2. PS.36:1 | 3. PS.42:6 |
| Sphingomyelin | 1. SM .d18:1/24:0 2. SM .d18:1/26:0 3. SM .d18:1/28:0 4. SM.d18:1/22:0 5. SM.d18:1/22:1 6. SM.d18:1/24:1 | 7. SM.d18:1/24:2 8. SM.d18:1/26:1 9. SM.d18:1/26:2 10. SM.d18:1/28:1 11. SM.d18:1/28:2 |
| Sphingosine | 1. Sphingosine.24:0. / sphinganine.24:1 | 2. Sphingosine.24:1 |
| Triglycerides | 1. TG.42:0 2. TG.42:1 3. TG.42:2 4. TG.42:3 5. TG.44:0 6. TG.44:1 7. TG.44:2 8. TG.44:3 9. TG.46:0 10. TG.46:1 11. TG.46:2 12. TG.46:3 13. TG.48:0 14. TG.48:1 15. TG.48:2 16. TG.48:3 17. TG.48:4 18. TG.48:6 19. TG.50:0 20. TG.50:1 21. TG.50:2 22. TG.50:3 23. TG.50:4 24. TG.50:5 25. TG.50:6 26. TG.50:7 27. TG.52:1 28. TG.52:2 | 29. TG.52:3 30. TG.52:4 31. TG.52:5 32. TG.54:1 33. TG.54:2 34. TG.54:3 35. TG.54:4 36. TG.54:5 37. TG.54:6 38. TG.54:7 39. TG.54:8 40. TG.56:1 41. TG.56:2 42. TG.56:3 43. TG.58:1 44. TG.58:2 45. TG.58:3 46. TG.58:4 47. TG.58:5 48. TG.58:6 49. TG.60:1 50. TG.60:2 51. TG.60:3 52. TG.60:5 53. TG.60:6 54. TG.62:2 55. TG.62:3 56. TG.62:4 |
